# Artificial Action Potential and Ionic Power Device Inspired by Ion Channels and Excitable Cell

**DOI:** 10.1002/advs.202301037

**Published:** 2023-04-07

**Authors:** Jung‐Soo Kim, Jongwoon Kim, Jinchul Ahn, Seok Chung, Chang‐Soo Han

**Affiliations:** ^1^ Institute of Advanced Machinery Design Technology Korea University 145 Anam‐ro, Seongbuk‐gu Seoul 02841 Republic of Korea; ^2^ School of Mechanical Engineering College of Engineering Korea University 145 Anam‐ro, Seongbuk‐gu Seoul 02841 Republic of Korea; ^3^ KU‐KIST Graduate School of Converging Science and Technology Korea University 145 Anam‐ro, Seongbuk‐gu Seoul 02841 Republic of Korea

**Keywords:** action potential, Donnan effect, excitable cell, ion channel, monovalent ion selectivity, neuron, power source

## Abstract

In vivo, the membrane potential of the excitable cell working by ion gradients plays a significant role in bioelectricity generation and nervous system operation. Conventional bioinspired power systems generally have adopted ion gradients, but overlook the functions of ion channels and Donnan effect to generate efficient ion flow in the cell. Here, cell‐inspired ionic power device implementing the Donnan effect using multi‐ions and monovalent ion exchange membranes as artificial ion channels is realized. Different ion‐rich electrolytes on either side of the selective membrane generate the ion gradient potentials with high ionic currents and reduce the osmotic imbalance of the membrane. Based on this device, the artificial neuronal signaling is presented by the mechanical switching system of the ion selectivity like mechanosensitive ion channels in a sensory neuron. Compared with reverse electrodialysis, which requires a low concentration, a high‐power device with ten times the current and 8.5 times the power density is fabricated. This device activates grown muscle cells by increasing power through serial connection like an electric eel, and shows the possibility of an ion‐based artificial nervous system.

## Graphical abstract

Cells generate an electric potential without a difference in total concentration due to the opposing concentration gradients of various ions and the selectivity of ion channels across the membrane. By mimicking the cellular system called the double‐Donnan effect, a novel nerve signal generation and high‐performance salinity gradient power system containing only high‐concentration electrolytes is realized.

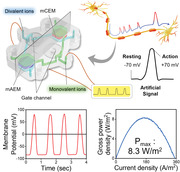

## 1 Introduction

**Figure 1:**
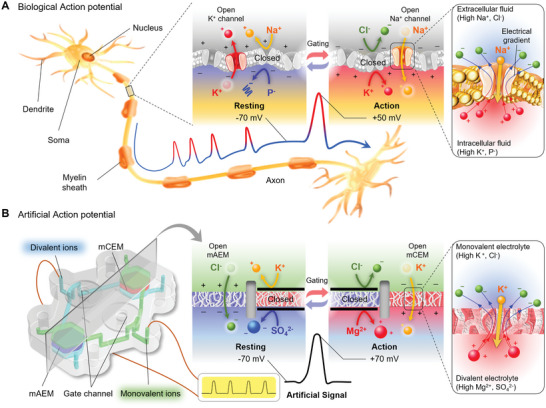
Schematic and mechanism of action potential in biological neuron and artificial chip. A) The basic structure of the neuron and generation of action potential. Stimuli or signals received from dendrites are transmitted to the axon. The cell membrane maintains negative potential during resting due to high potassium ion permeability through open potassium channels. Many intercellular potassium ions (K^+^) are in electrochemical equilibrium due to the Donnan effect caused by the extracellular sodium ions (Na^+^) and intracellular anionic proteins (P^−^) that do not permeate the transmembrane. This electrochemical equilibrium is maintained by the concentration gradient of permeable potassium ions and the electrical gradient expressed by the potential of −70 mV. The opening of the sodium ion channels by stimulation provides high permeability of sodium ions, and the relatively low permeability of potassium ions inside the cell and chloride ions (Cl^−^) with low permeability outside the cell form a new electrochemical equilibrium in the opposite direction, called the double‐Donnan effect. In this process, an action potential peak occurs. B) The artificial action potential chip consists of two membranes in parallel to realize the resting potential and action potential as the double‐Donnan effect. A high‐concentration electrolyte composed of monovalent ions fills the upper layer, and a high‐concentration electrolyte composed of divalent ions fills the lower layer. The monovalent anion exchange membrane (mAEM) causes the Donnan effect on the chloride ion, which is a monovalent anion, due to the impermeable cations and divalent anions. Conversely, in the monovalent cation exchange membrane (mCEM), a new Donnan effect on potassium, which is a monovalent cation, occurs due to the impermeable anions and divalent cations. This double‐Donnan system only has ion‐rich electrolytes and generates reversible electrical potential through channel gating.

Organisms generate electrical signals driven by ions not electrons.^[^ ^1^, ^2^ ^]^ In particular, excitable cells, such as neurons and muscle cells, generate and transmit potential. In vivo, ions travel through the cell membrane, and the ion channels in the membrane permeate or block specific ions to control ion flow and generate an electrical potential (**Figure** **1**A).^[^ ^3^, ^4^, ^5^, ^6^, ^7^, ^8^ ^]^ In the cell mechanism, various ions (K^+^, Na^+^, Cl^−^, etc.) and charged biomolecules are necessary, because different concentration of ions at the intra‐ and extracellular membranes produce resting potential and minimize the osmotic pressure, and maintain the balance in the electrophoretic and diffusive forces based on the ion gradients, the so‐called Donnan effect (Figure S1, Section S1, Supporting Information).^[^ ^9^, ^10^, ^11^, ^12^, ^13^, ^14^, ^15^ ^]^ Excitable cells generate resting and action potentials through the Donnan effect.^[^ ^15^, ^16^ ^]^ The gated‐ion channels alter the ion selectivity of the membrane, resulting in another Donnan effect (double‐Donnan) and electrical potential, called the action potential which is the electrochemical signal in an ion‐driven biological system.^[^ ^4^ ^]^ The action potential is positive and clearly distinct from the resting state with a negative potential. The living body generates information and communicates rapidly with these two opposite potentials generated by ions, and some organics, such as electric eels, emit large amounts of electrical energy.^[^ ^16^, ^17^, ^18^, ^19^, ^20^, ^21^ ^]^ Recently, various studies on artificial nerves and ion‐driven bioelectricity have been attempted, and they can be utilized in the development of biocompatible, wearable, and implantable sensor systems and energy devices as well as the replacement and rehabilitation of damaged organics.^[^ ^22^, ^23^, ^24^ ^]^

Here, we introduce a fully ion‐driven artificial spiking device and power source by mimicking the ion‐driven mechanism of the neuron (Figure 1B). It produces resting and action potential based on the double‐Donnan effect by the multi‐ion gradients and permselective membranes. Unlike osmotic power generation, this system, which consists of only concentrated electrolytes, has also been developed as a high‐power device.

The reported bioinspired ion‐gradient systems are based on reverse electrodialysis.^[^ ^22^, ^25^, ^26^, ^27^ ^]^ They generate an electrical potential by passing only cations or anions from a high‐concentration electrolyte to a low‐concentration electrolyte. However, their electrical performance is poor because of the low‐concentration electrolyte with low conductivity.^[^ ^22^ ^]^ Although attempts have been made to maximize the ion transport in membrane by using the nanopore technology, it is difficult to use on a large scale, and a large current loss in the low‐concentration electrolytes still remains.^[^ ^28^, ^29^, ^30^ ^]^ In contrast, in the living body, the total ion concentration of intracellular and extracellular fluids are similar (Figure S1 and Table S1, Supporting Information). Despite this similar concentration condition, the gradient of each ion and the membrane selectivity for a particular ion allow neurons to smoothly generate and conduct electrical potentials. Potassium ions (K^+^) and sodium ions (Na^+^), which play major roles in cell physiology and nerve signal generation, are contained in high concentrations inside and outside the cell, respectively.^[^ ^5^, ^6^, ^7^ ^]^ In the resting state, potassium ions, which are abundant inside the cell and are permeable to the extracellular fluid through resting potassium channels, form a negative resting potential (−70 mV).^[^ ^12^ ^]^ Conversely, voltage‐gated sodium channels open and sodium ions predominantly permeate into the intracellular fluid, generating a positive action potential (+50 mV).^[^ ^2^ ^]^ A living body controlling these two ions, which have the same polarity and opposite concentration gradients, generates electrical potentials even under similar total concentrations on both sides of the cell membrane, whereas conventional reverse electrodialysis controls ions with opposite polarity and same concentration gradients. In addition, other charged particles such as chloride ions (Cl^−^) and anionic proteins (P^−^), which have low permeability, support the generation of electrical potential and electroneutrality with low concentration difference between the two sides. It enables high electrical conductivity in all regions and high electrochemical stability with low osmotic pressure owing to similar total ion concentrations.^[^ ^13^, ^14^, ^15^, ^16^ ^]^

The proposed ionic system generates electrical potentials even at similar total ion concentrations by mimicking the ion composition and permselectivity of the excitable cell. Two ion‐rich electrolytes containing ions of different valences and the same polarity were separated by monovalent cation exchange membrane (mCEM) or monovalent anion exchange membrane (mAEM) to generate the Donnan potential in which the monovalent ion gradient is predominant, as shown in the right side of Figure 1B. In the artificial action state, membrane‐permeable potassium ions are affected by the electrical potential generated by the surrounding ions, including impermeable chloride ions and magnesium ions (Mg^2+^), thereby satisfying the electroneutrality of each electrolyte. In artificial resting state, ions, including impermeable potassium ions and sulfate ions (SO_4_^2−^), also generate opposite potentials against the gradient of permeable chloride ions. Similar to neurons that maintain resting and generate action by gated‐ion channels, a microfluidic chip with a mechanical gated‐ion channel that changes ion selectivity generates artificial nerve signals driven by ions. In addition, unlike the conventional ion‐gradient system, this system generates a high ionic current because both sides of the membranes contain only ion‐rich electrolytes, such as cells. Therefore, we designed a thin power device with membranes connected in series to obtain high voltages and currents.

## 2 Artificial Resting Potential Mimicking Donnan Effect of Cells

**Figure 2:**
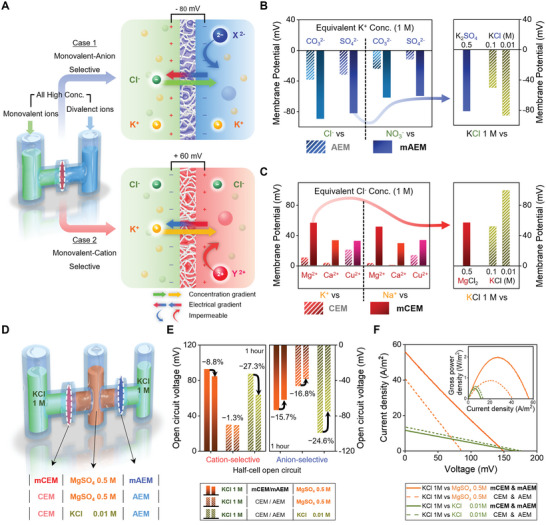
Artificial resting potential based on Donnan effect. A) Basic configuration for implementing the Donnan effect. Based on the monovalent exchange membrane, each reservoir contains high concentrations of monovalent and divalent ions. In the case of a monovalent anion exchange membrane, the same concentration of cations on both sides has no effect on the generation of membrane potential. Unlike impermeable divalent anions, high permeability monovalent anions cause the collapse of electroneutrality in both reservoirs. The ions in both reservoirs form an ionic layer around the membrane, which generate an electrical gradient that opposes the monovalent anion gradient. The same effect occurs in the case of the cation in the figure below. B) Donnan potential by several divalent anions for the common anion exchange membrane (AEM) and monovalent anion exchange membrane (mAEM). Even in the common anion exchange membrane, a weak membrane potential occurs due to the low ion mobility of divalent anions, and in the monovalent anion exchange membrane, this membrane potential is amplified. The right side shows the membrane potentials compared to those of reverse electrodialysis generated only with a single anion. C) Donnan potentials by several divalent cations for the common cation exchange membrane (CEM) and monovalent cation exchange membrane (mCEM). The right side shows the membrane potentials compared to those of reverse electrodialysis generated only with a single cation. D) Donnan system using an MgSO_4_ electrolyte containing only divalent ions to use two membranes together, and a comparison with a 100‐fold KCl (molar ratio of electrolytes) reverse electrodialysis system. E) Membrane potential maintenance performance of the Donnan system and reverse electrodialysis in an open circuit for 1 h. F) The *i*–*v* curve and gross power density (inner graph) of the Donnan system and comparison with 100‐fold KCl reverse electrodialysis.

**Figure** **2**A shows the details of the generation of an electrical potential based on the ion gradient without a low concentration. The cell membrane has ion channels that selectively permeate sodium or potassium ions which are similar in size and charge.^[^ ^3^, ^31^, ^32^ ^]^ However, to date, engineered ion‐exchange membranes for separating similar ions, such as biological ion channels, have not been reported. Commercial monovalent ion‐exchange membranes and multivalent ions were used to solve this problem.^[^ ^33^ ^]^ Divalent ions around monovalent ion exchange membranes behave like sodium ions, which do not permeate the cell membrane during rest. Therefore, the right reservoir of Figure 2A, which must have been a low‐concentration electrolyte in reverse electrodialysis, contained many divalent ions, so that there was no concentration restriction. The two cases on the right side of Figure 2B show systems with two opposite potentials based on this mechanism according to the cation and anion selection. We generated membrane potentials with a divalent electrolyte (0.5 m) and a monovalent electrolyte (1 m), which have the same number of charges at high concentrations (Figure 2B,C left). The two reservoirs in this system have the same number of counter‐charged ions; therefore, the membrane potential is generated by the selected monovalent ions. Divalent ions are impermeable; therefore, the opposite potential due to their gradient is weak. Using common anion exchange membranes (AEM) and cation exchange membranes (CEM) without monovalent selectivity, only a small potential is formed owing to the large size and low mobility of divalent ions. Instead of these membranes, the monovalent anion exchange membrane and cation exchange membrane generate large potentials similar to those of the biological membrane and of the conventional reverse electrodialysis based on KCl concentration gradients (Figure 2B,C right).

To enable the two membranes to function together, we redesigned the three‐reservoir system with two KCl 1 m electrolyte on both sides and a central reservoir containing only divalent ions, MgSO_4_ 0.5 m (Figure 2D). Compared to the reverse electrodialysis system in which the central reservoir contains 0.01 m KCl, this system with a common ion exchange membrane has little potential. However, when we used a monovalent ion‐exchange membrane, a potential similar to that of a reverse electrodialysis system was obtained. In addition, the membrane potential was more stable because of the lower osmotic pressure and lower water flow than in conventional reverse electrodialysis (Figure 2E and Figure S2, Supporting Information). As shown in Figure 2F, this system has four times higher current density and gross power density, with similar membrane potentials. Through comparison with the other cases in Figure 2F, the strongest performance is exhibited in the Donnan system composed of both a monovalent ion selective membrane and divalent ions. This is because the conductivity is significantly increased by replacing the low‐concentration electrolyte with a high‐concentration divalent ion (Table S2, Supporting Information). In particular, the difference in conductivity was clearly observed as the distance between the membranes increased (Figure S3, Supporting Information). In typical ion‐gradient systems, the low‐concentration section of high resistance increases and the discharging current rapidly decreases, whereas in this system, a high current can be confirmed even at a long electrolyte distance (Figure S4, Supporting Information). In addition, the charge capacity can be improved because of the large number of ions. Therefore, when this system is used as an energy device, both the power density and energy density can be significantly improved compared with conventional reverse electrodialysis (Figure S5, Supporting Information). Furthermore, low osmotic pressure makes the system electrochemically and mechanically stable.

## 3 Artificial Action Potential Based on Double‐Donnan System

Neurons use ion channel gating to switch between a state of high potassium ion permeability (resting) and state of high sodium ion permeability(action).^[^ ^5^, ^6^, ^7^ ^]^ The actuation of voltage‐gated sodium and potassium ion channels performs signal transduction that regenerates voltages in response to a changed potential at the front of the axon.^[^ ^3^, ^8^, ^17^ ^]^ In other cells, gated‐ion channels respond to various stimuli depending on the cell type. In sensory organs related to touch, mechanosensitive ion channels generate electrical potentials in response to mechanical stimuli.^[^ ^34^, ^35^ ^]^

**Figure 3:**
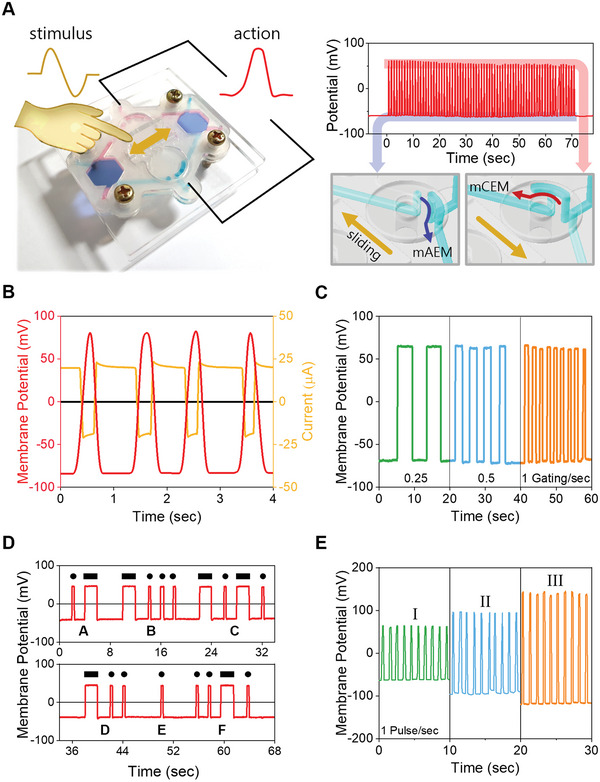
Artificial action potential based on double‐Donnan effect. A) A photograph of an artificial action potential chip and a stimulus–response mechanism. The channel is gated by the sliding bar that accepts stimuli in the center of the chip, and the membrane potential is generated according to the channel gating. B) Artificial action potentials and active ionic currents. C) Frequency change of the artificial action potential according to the gating rate. D) Generating Morse code A–F signals using long and short durations of action potential. E) Action potential amplification according to the series connection of action potential chips. Roman numerals are the number of connected chips.

To generate signals, such as in the nervous system, we fabricated an artificial action potential chip consisting of monovalent cation and anion exchange membranes in parallel and an artificial gated ion channel structure in a microfluidic chip (**Figure** **3**A). Monovalent ions are included in the upper layer of the two membranes, and only divalent ions are included in the lower layer, thereby generating opposite potentials. The artificial gating structure is composed of a sliding bar that receives an external stimulus and two rotating bodies that convert the stimulus into the gating of the fluid channels (Figure S6, Supporting Information). Under the rotating body, two channels connected to each membrane are arranged at a wider distance than the hole of the rotating body; therefore, its rotation opens only one channel while simultaneously blocking the other. Figure 3B shows the resting potential in which the channel connected to the monovalent anion exchange membrane is open, and the action potential generated by switching the channel connected to the monovalent cation exchange membrane using the gating structure.

The action potential generated by voltage‐gated channels of neurons is generally well known, but the living body generates various signal shapes depending on their roles.^[^ ^18^ ^]^ Some receptors in sensory organs generate long‐lasting potentials with slow adaptation to accept stimuli.^[^ ^36^ ^]^ In cardiac contractile cells, there is a plateau phase in which the action potential is maintained by a calcium ion gradient, which results in a ventricular contraction called excitation‐contraction coupling.^[^ ^37^ ^]^ An artificial action potential chip can generate various signals depending on the information generated or transmitted. Similar to the Morse code in Figure 3C,D, artificial action potentials can be transmitted with specific information by controlling the duration and space of the signal. In addition, when we reconnected several action potential chips in series, the signal was amplified by simultaneous activation, indicating the possibility of ionic communication similar to cells (Figure 3E). This implies that ion‐driven signals can be generated and transmitted in the form of coded data in a biological system.

## 4 High‐Power Device Using Multi‐Ion Gradient and Permselectivity

In terms of signal transmission, the action potential is transmitted as a chain action along the membrane of the axon, and at the myelin‐insulated membrane, it is conducted directly as an ionic current along the inside and outside of the cell, called saltatory conduction.^[^ ^38^ ^]^ In this mechanism, ions directly transmit signals more rapidly and efficiently than the chained activation of the non‐myelinated axon membrane.^[^ ^39^ ^]^ In addition, cell‐to‐cell signal transmission is also accomplished by ionic conduction, such as in the gap junction of the cardiac cells, where fast synchronization is required.^[^ ^40^ ^]^ This type of action is possible because the fluid inside and outside the cell is rich in ions, demonstrating the possibility of our system for efficient signal transduction with high conductivity (Figure S7, Supporting Information).

**Figure 4:**
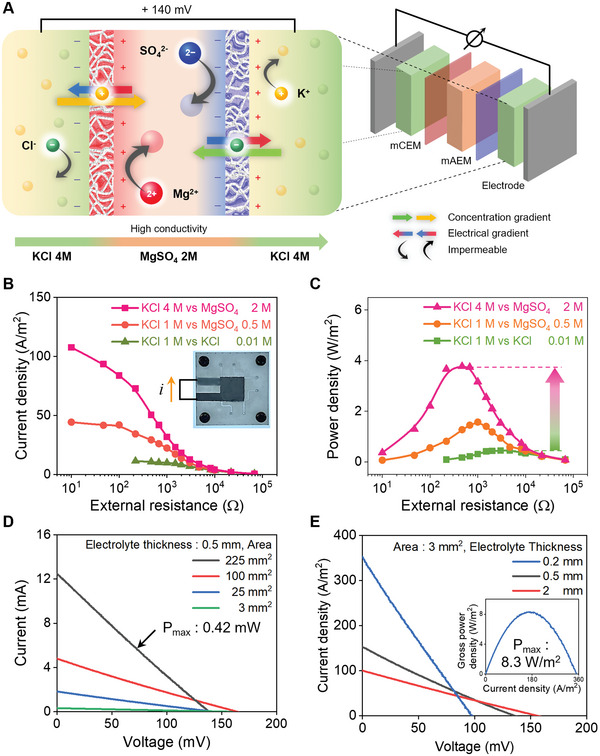
Power density and electrical performance of the ionic power devices. A) The structure of power device composed only of high‐concentration electrolyte. B) The current density of the Donnan systems (pink and orange) and comparison with the 100‐fold KCl gradient system (green) in the power device. At the resistance with the maximum output power, the current density of the Donnan system with saturated electrolytes was 50.5 A m^−2^. This is ten times the current density in the KCl gradient system, indicating that the improvement in conductivity is the most important factor in the power density. The inner photograph is optimized power device. Small pores and channels are the entrances and exits of the electrolytes. The dark gray area in the device represents the Ag/AgCl electrode located at the top and bottom. C) The power density of the Donnan systems (pink and orange) based on different concentrations of electrolytes, and a comparison with the 100‐fold KCl reverse electrodialysis (green). The maximum power density is 3.8 W m^−2^ with electrolytes at saturated concentrations and commercial monovalent ion exchange membranes. The maximum power density is generated when the external and internal resistances are equal. D) The *i*–*v* curve of the optimized power device in various areas. The maximum power of the 225 mm^2^‐sized device is 0.42 mW. E) The *i*–*v* curve of the power device in various electrolyte thickness, the maximum power of the 0.2 mm‐thickness device with 3 mm^2^ area is 8.3 W m^−2^.

For the same purpose, an artificial potential system conducts electrical signals through high‐conductivity channels containing many ions. We designed a new power device in which two high‐concentration electrolytes with thicknesses of 2 mm and two membranes were connected in series, as shown in **Figure** **4**A. Two impermeable ions in each membrane induce a double‐Donnan effect. In addition to the double‐Donnan effect, ion‐rich electrolytes with low resistance to ion flow provide efficient electrical performance. A maximum power density of 3.8 W m^−2^ with a high current density of up to 100 A m^−2^ was obtained at a saturated concentration (Figure 4B,C). This is 8.5 times the power density with ten times the current density compared to the case of configuring it in a 100‐fold KCl gradient. Even when a high concentration of the KCl gradient system is raised to saturation, there is a limit due to the trade‐off between the low‐concentration conductivity and the membrane potential (Figure S8, Supporting Information). A more optimized power device with an electrolyte thickness of 0.5 mm and a large area generates a high current of up to 12 mA in a size that can be used as a battery (Figure 4D). The maximum power of this single device was 0.42 mW. In addition, the power device scaled down to electrolytes with thickness of 0.2 mm generates a maximum power density of 8.3 W m^−2^ (Figure 4E). Under the similar level of membrane performance, conventional LED cannot overcome the Donnan system containing only high concentrations (Figure S9, Supporting Information).

**Figure 5:**
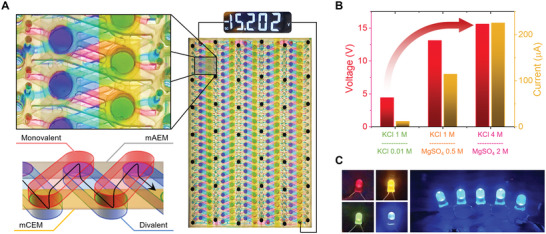
High‐power device with spirally connected structure. A) Full and close‐up photos, and schematics of the A4‐sized high‐power device. Based on a layer in which two long‐cut membranes are arranged in parallel, it is a series structure in which two electrolytes are alternately connected in a spiral. B) Comparison of the voltage and current of the high‐power device in the Donnan system and 100‐fold KCl reverse electrodialysis. C) High‐power device connected to LED. The rated voltage of the LEDs used was 2.0–2.2 V, and the rated current was 20–40 mA. The four pictures on the left show the red, yellow, green, and blue LEDs connected. On the right, five blue LEDs are connected in series and turned on at maximum brightness.

Electric eels generate high voltages through a series of membrane potentials.^[^ ^20^, ^22^ ^]^ For this to be released with high power without electrical loss in vivo, the Donnan effect must be supported so that the intracellular and extracellular fluids that conduct the membrane potentials are rich in ions. We designed a thin and wide series‐connected high‐power device by placing two ion‐exchange membranes in parallel on a plane and spirally connecting two electrolytes above and below them, as shown in **Figure** **5**A. The high‐power device was 2 mm thick and A4‐sized. The spiral structure connects the membrane potentials 270 times in series with only ten membranes. Thin structures such as those in this study require long electrolyte lengths to connect multiple membranes located on a single plane. With reverse electrodialysis, long and low‐concentration electrolytes having a large resistance waste voltage and generate a low ion current.^[^ ^22^ ^]^ The Donnan system, where all electrolytes are ion‐rich, minimizes this electrical loss, and emits 15.2 V and 225 µA, which can turn on five series‐connected LEDs (Figure 5B,C).

**Figure 6:**
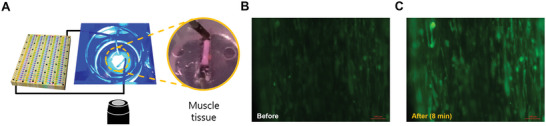
Muscle stimulation using a high‐power device. A) Schematic diagram of the muscle stimulation experiment. Fluorescence imaging of a calcium ion flux in the excitation–contraction coupling process of muscle generated by the power supply of the high‐power device. B,C) Change in the calcium fluorescence image in the muscle tissue before and after stimulating for 8 min.

We applied Donnan potential‐based stimulation to muscle tissue using this high‐power device (**Figure** **6**A). Neuronal stimulation (neurotransmitters) induces depolarization of muscle cells and generates muscle contractions through an excitation–contraction coupling mechanism which is a voltage‐ and calcium‐dependent process.^[^ ^41^ ^]^ In muscle cells, stimulation by acetylcholine opens ion channels and generates an action potential. The action potential is transmitted as a signal and also opens the calcium channel to induce the influx of calcium ions to activate the Ca^2+^ signaling pathway for muscle contraction. To characterize the muscle tissue before application of our ion‐based nerve system, we performed immunostaining and calcium imaging with acetylcholine chloride treatment which is an endogenous neurotransmitter that binds to the acetylcholine receptor of the muscle cell membrane (Section S2, Supporting Information). The skeletal muscle cells in the 3D tissue expressed myosin heavy chain type II and striped pattern of sarcomeric alpha‐actinin at seven days of differentiation. At this time point, the muscle cells showed an increase in intracellular calcium ions in response to acetylcholine chloride (Figure S10, Supporting Information). Since the direct initiation of the Ca^2+^ signaling pathway is an action potential, we have activated the muscle tissue with Donnan system that emits sufficient potential and power by controlling ions. A single action potential chip was insufficient to activate the muscle tissue in which several hundred cells were gathered. However, a high‐power device with multiple membranes successfully activated muscle tissue. A distinct change was observed by comparing the fluorescence images before and after stimulation (Figure 6B,C). Through calcium fluorescence, it can be seen that the high‐power device performed sufficient electrical stimulation of many cells in the muscle tissue.

The artificial action potential chip and power devices use monovalent ion selectivity to separate ions of the same polarity. The monovalent ion selectivity of commercial membrane is sufficient to generate as much biological membrane potential as possible, even in the range of total ion concentration in the living body (Figure S11, Supporting Information). Above all, the ion current and power rise dramatically compared to the conventional reverse electrodialysis systems by replacing the low concentration with the high concentration. However, artificial system has larger gradients of each ion than that of the living body, because the membrane potential generated by the separation between multivalent ions is smaller than the potential generated by separation between monovalent ions (Table S1, Section S3, Supporting Information). In addition, multivalent ions have lower conductivity than monovalent ions in same concentration. Although we used a saturation concentration to maximize the electrical performance, the maximum concentration of multivalent ions was also lower than that of the monovalent ions. Therefore, when both electrolytes are used at their maximum concentrations, there is a limit to lowering the osmotic pressure. Ultimately, to secure the stability and higher performance of the system, it is necessary to improve engineered membranes that separate monovalent ions with the same polarity, such as biological ion channels. Since many membrane studies suggest excellent selectivity in the conventional ion gradient system, further development is expected by introducing the membranes into our multi‐ion concentrated system. If a selectivity of monovalent ion‐selective membrane similar to that of the current charge‐selective membrane is developed, not only excellent performance for the Donnan system, but also higher performance can be expected in the RED by selecting between divalent ions of low‐concentration and monovalent ions of high‐concentration with same charge (Figure S12, Supporting Information). Another important parameter for electrical performance is the interlayer spacing between the membranes. Because the area resistance of a unit cell depends on the thickness of the electrolyte, the denser the structure, the greater the power per unit area. Therefore, if membranes separating monovalent ions with the same polarity are developed and used in denser structures, this system becomes more similar to that of living organisms. In addition, artificial systems capable of using higher concentrations than biological cells are expected to achieve higher membrane potential and power density.

## 5 Conclusion

We implemented high‐power device and artificial action potentials by controlling the flow of ions such as neurons. In particular, electrical potentials are generated by multi‐ion gradients with only ion‐rich electrolytes by mimicking the ionic composition and selectivity of a living body, which is called the double‐Donnan effect. This system emits a higher current and power owing to its high electrical conductivity compared to that of conventional ion gradient systems. The use of ion gradients without concentration restrictions suggests a new performance‐enhancing method for osmotic power generation. Furthermore, this system, which can control ion flow and electricity with a mechanism similar to a living body, has high utility in biofriendly technologies. It is expected to be available as an implantable and wearable energy device or artificial neural system.

## 6 Experimental Section

*Materials*: All electrolytes were aqueous solutions, and the dissolved salts were purchased from Sigma‐Aldrich. Neosepta ion exchange membrane family as an ion‐exchange membrane was purchased from Astom. CSE (for cations) and ASE (for anions), which have common charge selectivity, were used to implement conventional reverse electrodialysis as a control group. For the Donnan system, CIMS for monovalent cation permselectivity and ACS for monovalent anion permselectivity were used.

*Characterization of Artificial Resting Potential*: The ion exchange membrane was fixed to an H‐shaped cell. The two 2 mL glass reservoirs have a joint part and solution injection part, so that an H‐shaped cell is formed by combining the joint parts of the two reservoirs. A membrane supported by 0.5 mm silicone rubbers was fixed between the joint part with metal tongs. Ag/AgCl electrodes were used to measure the electrical properties, and salt bridges were used because the electrolytes were different, as shown in Figure S13 (Supporting Information). The salt bridge was made of agarose gel containing KNO_3_ 0.5 m and connected from the solution injection part of the H‐shaped cell to another chamber containing KCl 1 m electrolyte. By inserting an Ag/AgCl electrode into this chamber, the influence of the electrode potential owing to the concentration difference was excluded.^[^29^]^ A multimeter (Keithley 2002) was used to measure the potential. A three‐reservoir system with a +‐shaped glass reservoir added to the center fixes the two membranes connected in series. In the case of using two membranes, because both ends have the same electrolyte, Ag/AgCl electrodes were inserted directly on both sides without the use of salt bridges; thus, the electrical performance of the cell was completely secured without any electric loss from the salt bridge. The electrical performance of the three‐reservoir system with two membranes connected in series was confirmed using a multimeter (Keithley 2002) for potential measurement, and a potentiometer (VersaSTAT 3, Princeton Applied Research) for *i*–*v* curve measurement. Details of the osmotic pressure measurements are provided in Section S4 (Supporting Information).

*Artificial Action Potential Chip*: Based on the layer in which the two ion exchange membranes (CIMS, ACS) were positioned, a channel layer filled with MgSO_4_ 0.5 m electrolyte was positioned below. Another channel layer filled with KCl 1 m electrolyte and a gating structure were included at the top. The channel layers were fabricated using a 1 mm acrylic plate. It was fixed with 3 mm acrylic plates for sealing above the upper channel layer and below the lower channel layer. Silicon rubber (0.5 mm) was inserted between each layer. The channel structures of all the layers were machined by laser cutting. The gate structure was composed of a bar gear (sliding bar) that was exposed to the outside environment and received mechanical stimulation. Two circular gears (rotating bodies) with holes connected the channels. These two gears convert linear motion into rotational motion such as a rack and pinion. Two circular gears were connected on both sides of the bar gear so that they rotated in opposite directions, and the two holes included in each circle gear opened only the left or right side toward the same direction. All gating structures were fabricated by laser cutting of 1 mm acrylic plates. Ag/AgCl was used as the electrode, and a salt bridge was used for the channel filled with an MgSO_4_ 0.5 m electrolyte to exclude the electrode potential effect. The voltage and current were measured using a Keithley 2002 multimeter. For a higher scan rate, the chronoamperometry mode and chronopotentiometry mode of the VersaSTAT 3 potentiometer were also used. In each mode, the voltage and current of the measuring device were set as 0.

*Power Device with High Ion Strength*: For high power density, a power device was constructed by replacing the reservoir of the H‐shaped cell with an acrylic plate with a thickness of 1 mm. 0.5 mm silicon rubber was inserted between layers for sealing, so the total thickness of the electrolyte is 2 mm. The optimized power device was replaced with at least 0.2 mm silicone rubber, which served as both a sealant and reservoir (Figures S13 and S14, Supporting Information). The power density was calculated by measuring the current of the circuit connected to the standard resistor, as shown in and Figure S14 (Supporting Information). The maximum power of the optimized power device was calculated using the *i*–*v* curve. The details of the calculations are provided in Section S6 (Supporting Information). For high‐power device with a high voltage of A4 size, CIMS and ACS membranes of 300 × 10 mm^2^ sizes were alternately arranged. The top layer of the membrane is a channel layer containing a KCl electrolyte that connects the bottom left of the CIMS to top right of the ACS. The lower layer of the membrane is a channel layer containing an MgSO_4_ electrolyte, connecting the bottom right of the ACS to the top left of CIMS. Thus, the two channel layers connected the two membranes in a spiral structure. This was repeated to obtain a structure in which 270 membrane potentials were connected in series to ten membranes. The measuring devices were the same as those used for the artificial action potential chip.

*Imaging Calcium Transients of Tissue Engineered Muscle during Stimulation*: Details of the fabrication of the tissue‐engineered skeletal muscle are provided in Section S2 (Supporting Information). To capture the response of the skeletal muscle cells to artificial action potentials, intracellular calcium transients in the tissue‐engineered muscle bundle during stimulation were imaged with an artificial action potential chip. Briefly, tissue‐engineered human skeletal muscle bundles (minimally 8 d differentiated) were incubated with a 1:1 mixture of DMEM/F12 medium (Gibco) and Fluo‐4 Direct Calcium Assay Reagent (Invitrogen, F10471) for 30 min at 37 °C. After incubation, the loaded medium was replaced with the imaging buffer (137 × 10^−3^ m NaCl, 5.3 × 10^−3^ m KCl, 2 × 10^−3^ m CaCl_2_, 1 × 10^−3^ m MgCl_2_, 11.1 × 10^−3^ m d‐glucose, and 20 × 10^−3^ m HEPES). A pair of Ag/AgCl electrodes was placed in the muscle tissue culture chamber, and they were, respectively, connected to the Ag/AgCl electrodes at both ends of the high‐power device through wires. The calcium fluorescent transients were captured before and after stimulation at room temperature using a fluorescence microscope (Zeiss) with 10× magnification. The obtained images were analyzed using Zen software.

## Author Contributions

J.‐S.K. and C.‐S.H. conceived the project and designed the experiments. J.‐S.K. performed all experiments and analysis. J.K. helped characterize the ion exchange membranes. J.A. and S.C. helped in the muscle stimulation experiment. J.‐S.K. and C.‐S.H. wrote the manuscript.

## Conflict of Interest

The authors declare no conflict of interest.

## Supporting information

Supporting Information

## Data Availability

The data that support the findings of this study are available from the corresponding author upon reasonable request.
