# Supplementary material for: Artificial Action Potential and Ionic Power Device Inspired by Ion Channels and Excitable Cell
Source: Adv Sci (Weinh). 2023 Apr 7;10(16):2301037. doi: 10.1002/advs.202301037 (PMC10238195; doi:10.1002/advs.202301037)
Supplement: Supplementary file 1 — Supporting Information [file ADVS-10-2301037-s001.pdf]

ADVANCED SCIENCE
Open Access

# Supporting Information

for *Adv. Sci.*, DOI 10.1002/advs.202301037

Artificial Action Potential and Ionic Power Device Inspired by Ion Channels and Excitable Cell

*Jung-Soo Kim, Jongwoon Kim, Jinchul Ahn, Seok Chung and Chang-Soo Han**

Supporting Information

# Excitable Cell-inspired Artificial Action Potential and Power Device Using Double-Donnan System

*Jung-Soo Kim, Jongwoon Kim, Jinchul Ahn, Seok Chung, and Chang-Soo Han **

**Figure S1 | Biological membrane potentials**

Details are provided in Supporting Information 1. **(A)** Ion composition of intra and extra membrane in vivo **(B)** Maintaining the resting potential and Donnan effect of the high-permeability potassium ion gradient and the opposite electrical gradient of the membrane. **(C)** Another Donnan effect of sodium ions in action, called double-Donnan.

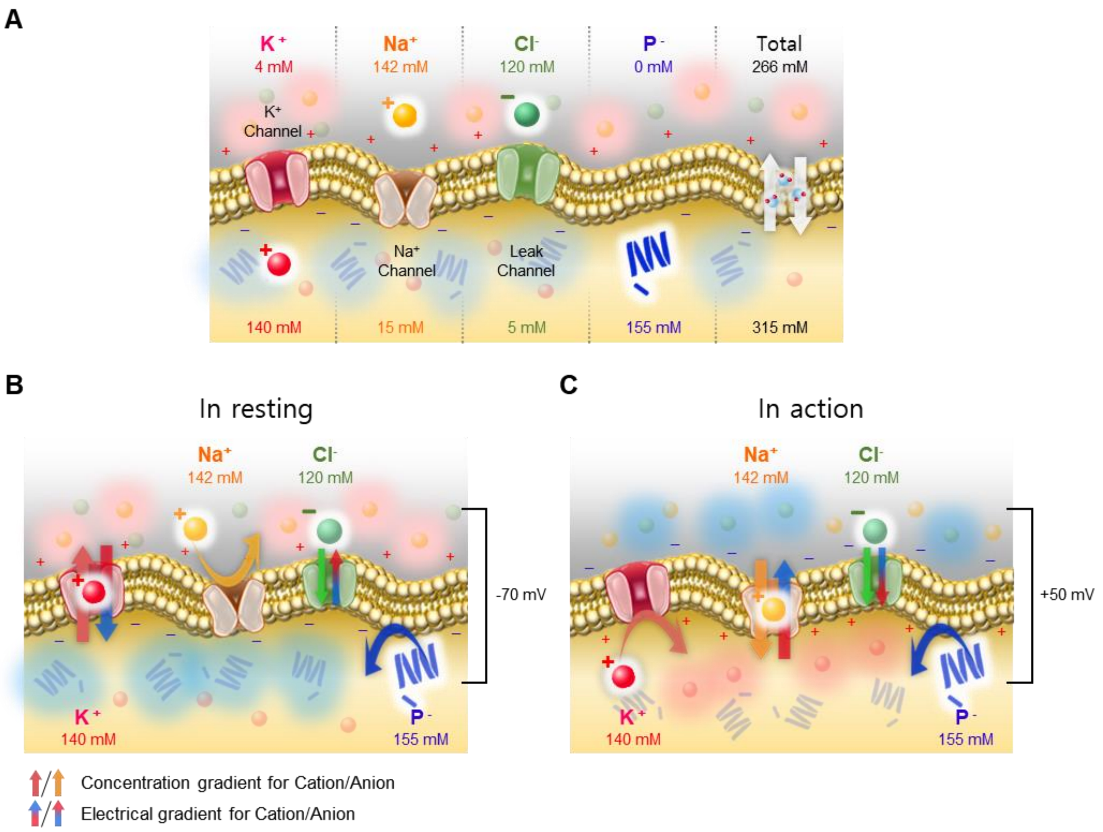
A
K+
4 mM
Na+
142 mM
Cl-
120 mM
P-
0 mM
Total
266 mM
K+ Channel
Na+ Channel
Leak Channel
140 mM
15 mM
5 mM
155 mM
315 mM
B
In resting
Na+
142 mM
Cl-
120 mM
-70 mV
K+
140 mM
P-
155 mM
C
In action
Na+
142 mM
Cl-
120 mM
+50 mV
K+
140 mM
P-
155 mM
Concentration gradient for Cation/Anion
Electrical gradient for Cation/Anion

**Figure S2 | Osmotic pressure of two electrolytes with cation exchange membrane.**

Experimental details are provided in Supporting Information 4. **(A)** The amount of water movement with respect to time was measured when the two electrolytes were in contact with an ion-exchange membrane. Each line represent a linear fit of the measurements. The smaller the total concentration difference, the lower the osmotic pressure. **(B)** Prediction of water flow in a small device for osmotic power generation. When the contact area with the membrane is maintained, the high osmotic pressure of the reverse electrodialysis system is concerned about the rapid exhaustion of the low-concentration electrolyte. **(C)** Prediction of membrane potential reduction by osmosis. The Donnan system keeps the membrane potential stable. These predictions assume a situation where there is no ion flow with a disconnected device without electrode insertion, and a more realistic rapid decrease with the discharge process including ion flow is illustrated in Figure S4. These results show that the Donnan system has better stability even in the case of an unused device.

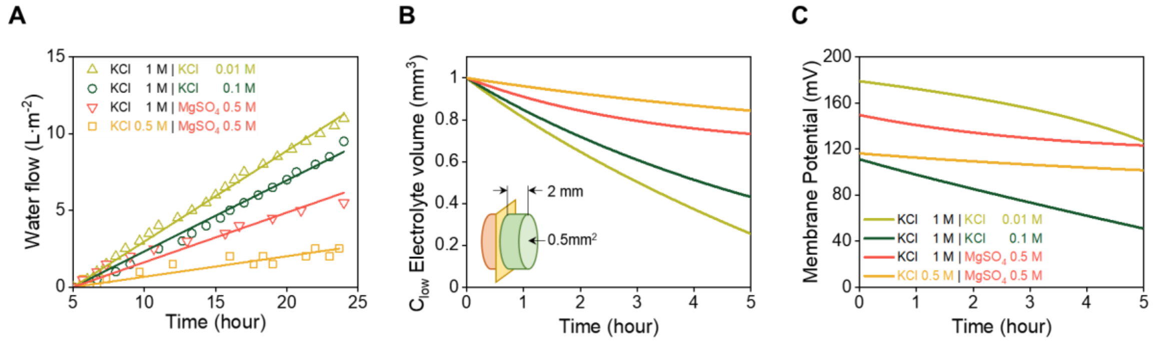

**Figure S3 | Discharging curve according to the electrolyte distance**

**(A)** Current density in the Donnan system as a function of divalent electrolyte length. **(B)** Current density of the reverse electrodialysis system along the length of the KCl 0.01 M electrolyte. The initial current is low owing to the KCl 0.01 M, which has low conductivity, and, over time, ions flow in and the current rises. However, it decreased again decreasing membrane potential. All reservoirs were open, so that water flowed by osmotic pressure. A narrow distance has a high maximum current, but the water flow caused a sharp decrease in the ion gradient and current density.

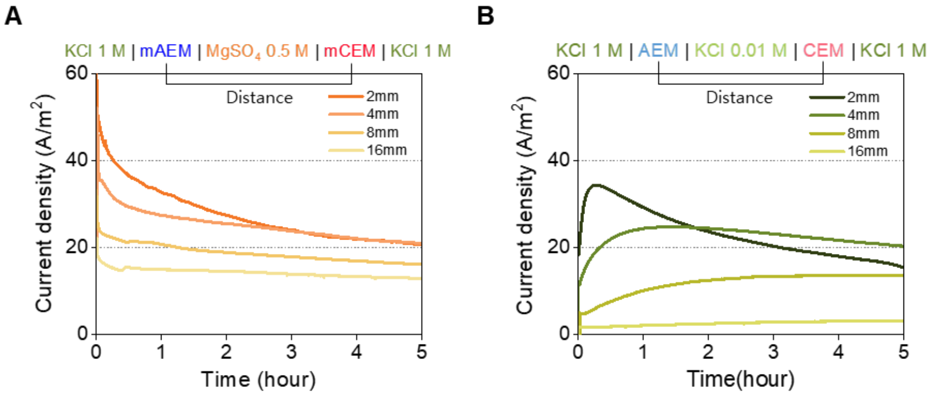

**Figure S4 | Theoretical analysis of the discharge process**

The details of the theoretical analysis process are in the Supporting Information 5. **(A)** Current density in the Donnan system as a function of divalent electrolyte length. For monovalent ion selectivity, 14, similar to the membrane used in the experiment, was applied. **(B)** The decrease in the concentration of initial ions in the two electrolytes and **(C)** membrane potential due to the ion flow accompanying the discharge. The reduced ions are added to the counter electrolyte, but are not plotted in this graph to observe the initial ion change. **(D)** Current density of the reverse electrodialysis system along the length of the KCl 0.01 M electrolyte. For charge selectivity, 40, similar to the membrane used in the experiment, was applied. **(E)** Concentration change of the two electrolytes and **(F)** membrane potential decrease due to the ion flow accompanying the discharge. When the length of the central electrolyte is long, the membrane potential and concentrations are well maintained, but this is because current cannot flow due to the high resistance of the central electrolyte. Therefore, sufficient power cannot be emitted.

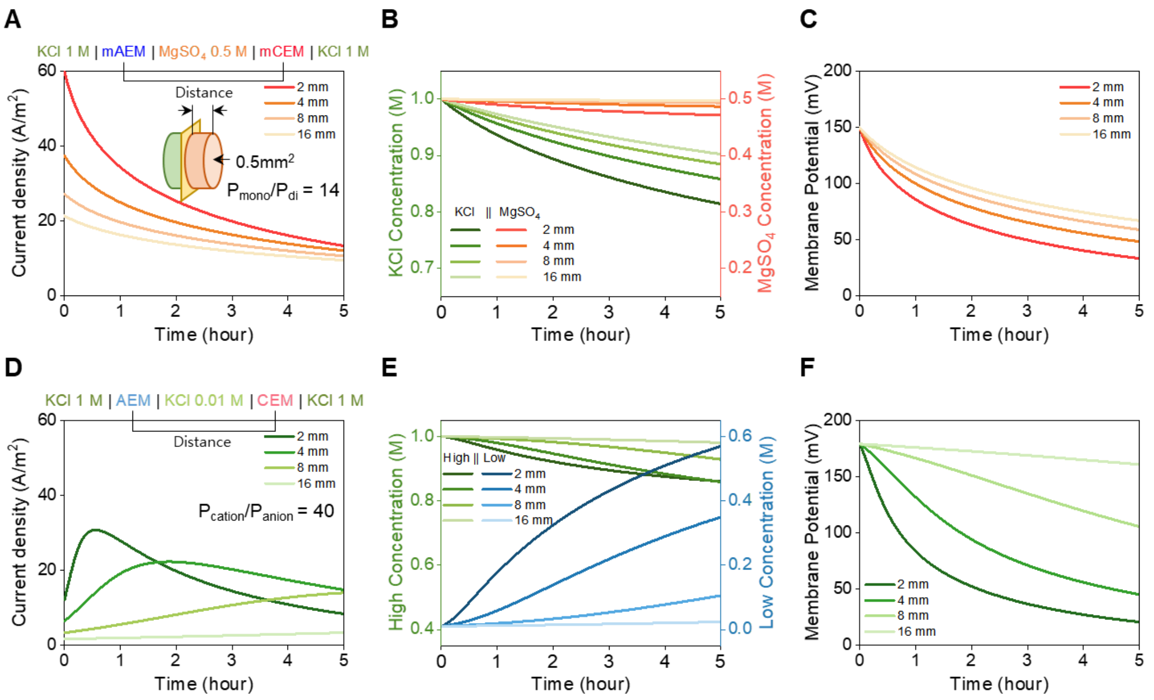

**Figure S5 | Energy density of the Donnan system**

The energy density was calculated from the current and voltage at the 2 mm length in Figure S4. The energy density at each time is a cumulative value, meaning the total amount of energy during each time. RED system (green) is KCl 1M vs KCl 0.01M, and Donnan System (red) is KCl 1M vs $MgSO_4$ 0.5M. In the same volume, the Donnan system has twice as much charge as the RED system, so it is about twice as high at similar membrane potentials and conductivities.

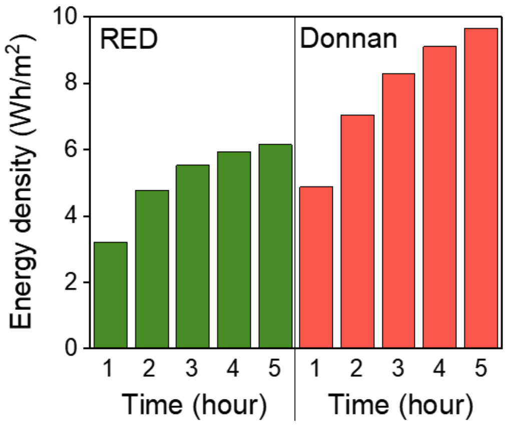

**Figure S6 | Mechanical structure of the action potential chip**

**(A)** Photograph and **(B)** details of the action potential chip. A central slide bar rotates the upper and lower gears in opposite directions, gating channels in the same direction. As only one of the left or right channels is opened, a membrane potential according to the selectivity of the corresponding membrane is generated. The selectivity and gating function of the biological membrane were mimicked by the membranes and gating structure in the action potential chip, respectively. **(C)** The state of a gate structure in the resting and action.

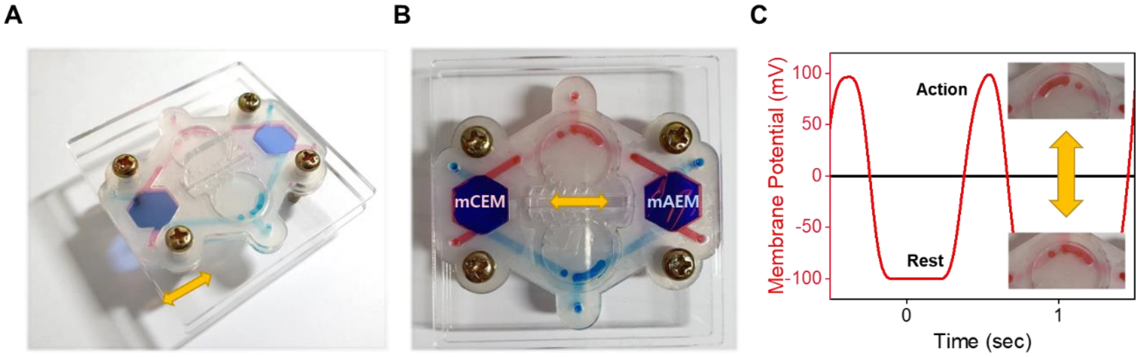

**Figure S7 | Power and conductivity of action potential chip for signal transduction**

**(A)** RMS current density and active power density of the current pulse emitted by the signal generation of the action potential chip. Active power density is the power density calculated as the RMS current density. The dotted line(KCl 100-fold) is the action potential chip composed of KCl 1 M vs KCl 0.01 M electrolyte and common ion exchange membranes. The solid line(Donnan) is an action potential chip composed of KCl 1 M vs $MgSO_4$ 0.5 M electrolyte and monovalent ion exchange membranes. Action signals must pass through electrolyte-filled fluidic channels, as shown in Figure S6 B. However, in the reverse electrodialysis configuration(KCl 100-fold), intact signal transmission is difficult due to low conductivity. The Donnan system configuration performs signal transmission at high power without these problems. **(B)** Signal transfer performance dependent on an external resistive load. The Donnan system exhibits clearly distinguishable signaling performance in a wider range of external environments.

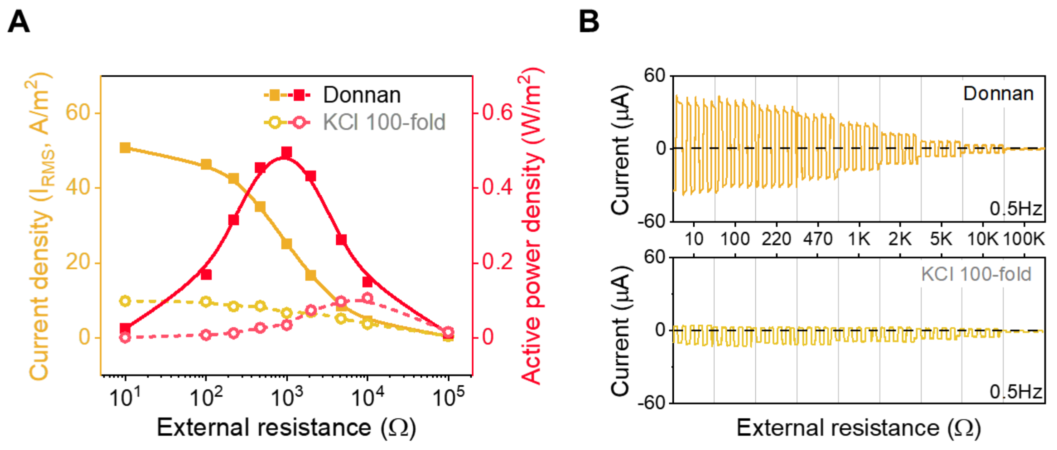

**Figure S8 | Power density of Donnan system in various electrolytes.**

**(A)** Power density of the Donnan system as a function of different $MgSO_4$ concentration in the power device. Between the two electrodes, KCl 4 M, monovalent cation exchange membrane, $MgSO_4$ electrolyte, monovalent anion exchange membrane, and KCl 4 M were connected in series. **(B)** Power densities of several reverse electrodialysis systems. Between the two electrodes, KCl 4 M, a common cation exchange membrane, a low-concentration KCl electrolyte, a common anion exchange membrane, and KCl 4 M were connected in series. Although 40-fold KCl has a lower membrane potential than 400-fold KCl, it has a higher power density owing to its high conductivity.

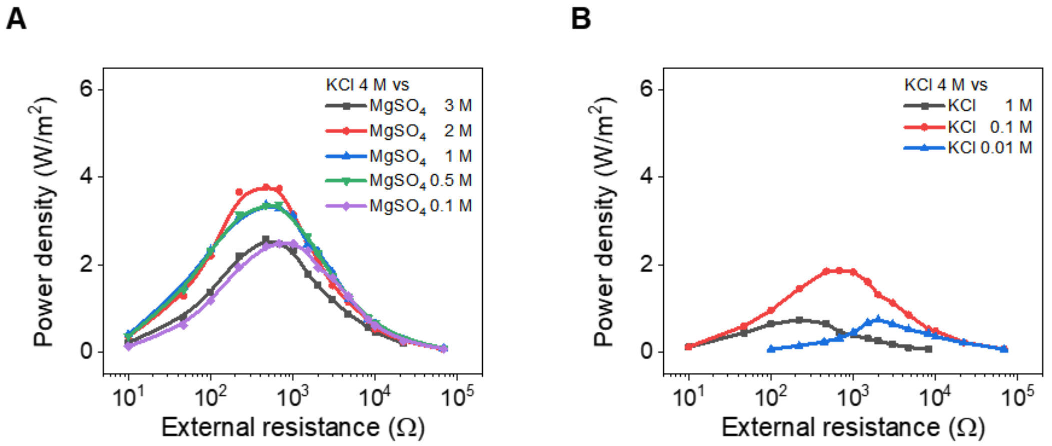

**Figure S9 | Power density of a power device based on the Donnan system.**

Based on the measured power density in Figure 4, the electrolyte resistance calculation in Table S2 and Figure S4 was introduced to show the predicted power density according to the thickness of the electrolyte. experimental values related to this is Experimental values related to this are indicated by light-colored symbols with borders.

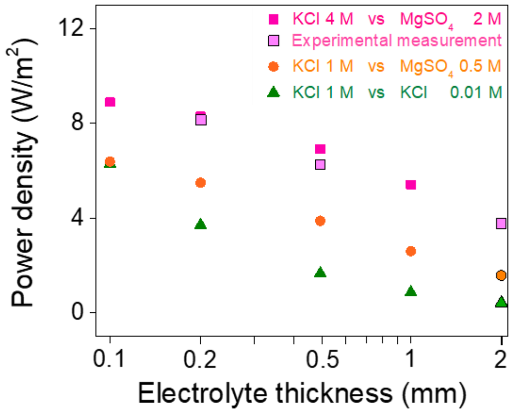

**Figure S10 | Characterization of tissue-engineered skeletal muscle bundle**

Experimental details are provided in Supporting Information 2. **(A)** Immunostained images of muscle cells at seven days of differentiation. **(B)** Change in calcium fluorescence in the muscle tissue before and after stimulation with 0.1 mM acetylcholine chlorides.

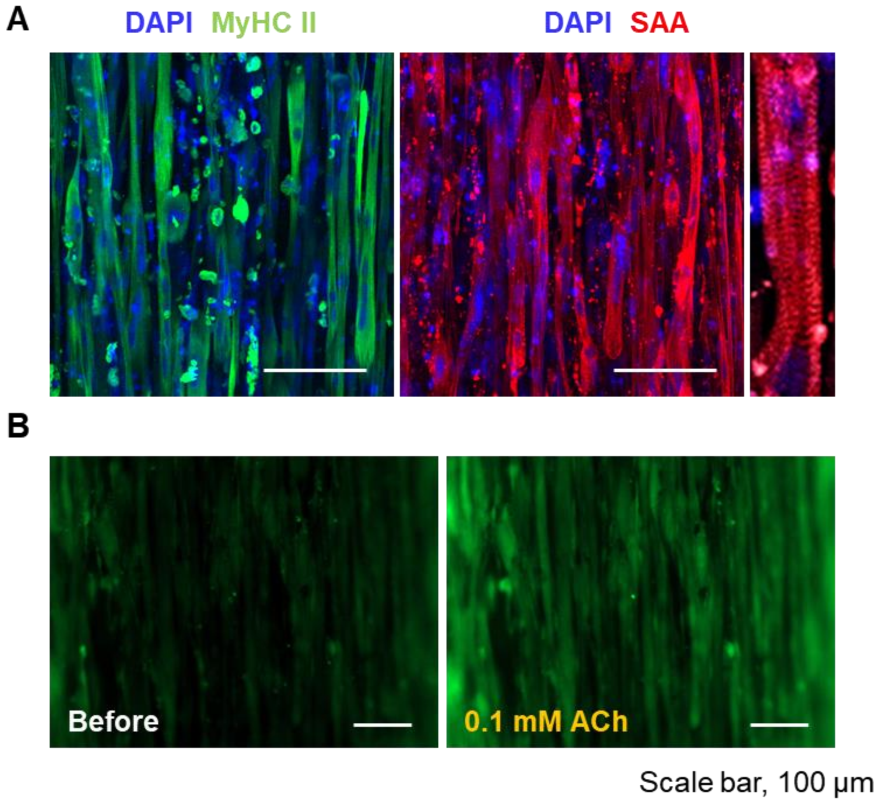

**Figure S11 | Membrane potentials in various concentration based on Donnan effect**

The Donnan system consists of a KCl electrolyte, monovalent cation exchange membrane, $MgSO_4$ electrolyte with half the concentration of KCl electrolyte, monovalent anion exchange membrane, and KCl electrolyte connected in series, as shown in Fig. 3A. That is, the KCl : MgSO4 concentrations of each dot in the graph are 4:2, 2:1, 1:0.5, 0.5:0.25, 0.25:0.12, 0.12:0.06, 0.06:0.03, and 0.03:0.015. The shaded area represents the concentration range of living organisms.

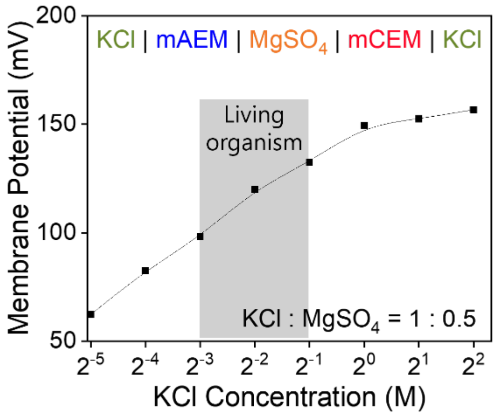

**Figure S12 | Theoretical membrane potential of Donnan and RED systems according to permselectivity and concentration.**

Details are in Supporting Information 3. The blue line is the ideal maximum membrane potential of the biological systems. Donnan system is as if the high-concentration electrolyte is in contact with the much lower-concentration electrolyte than RED system having same concentration difference, because the ions contained in the low-concentration electrolyte are endowed with impermeability. Therefore, the membrane potential of the Donnan system is higher if the permselectivity is the same.

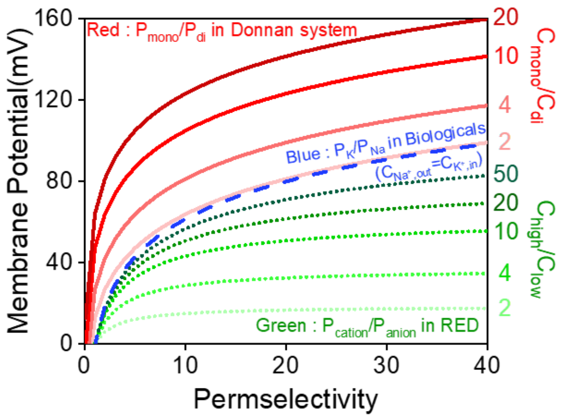

**Figure S13 | Schematics of experiments to measure electrical performance**

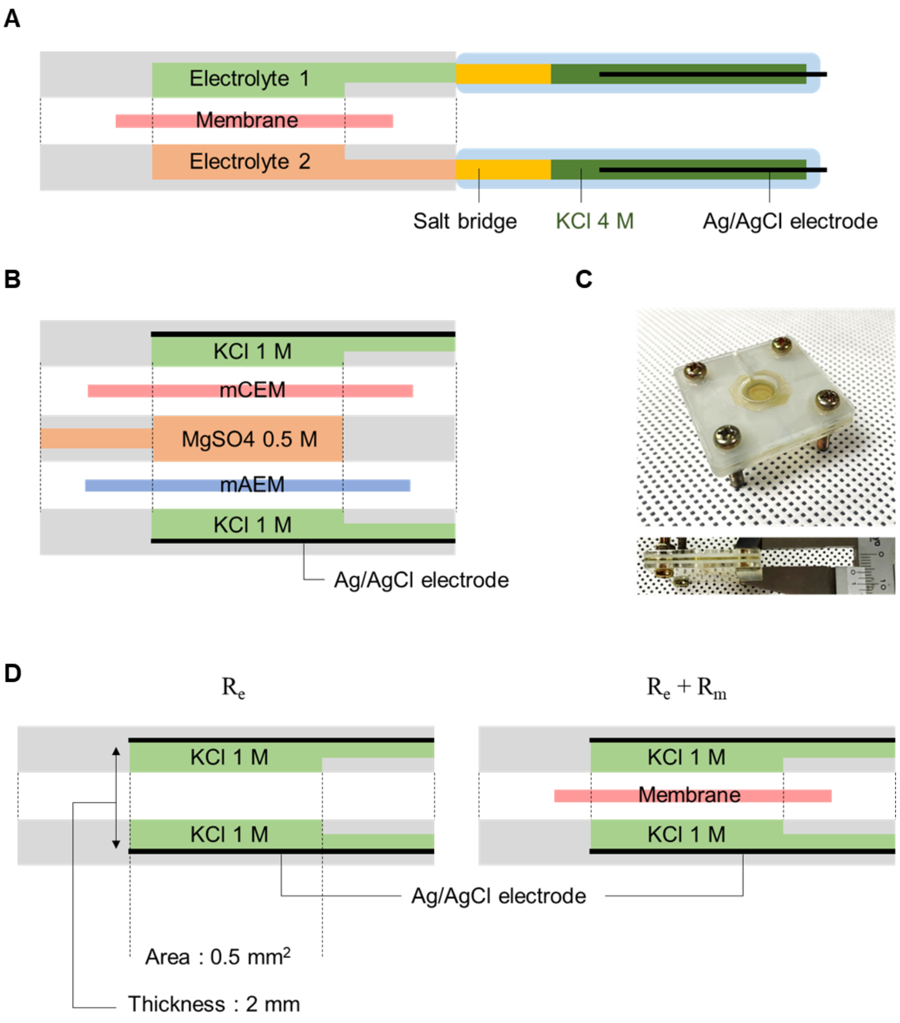


**(A)** A structure containing a salt bridge to measure the potential of a single membrane. **(B)** A schematic and **(C)** Photograph of a device based on Donnan system that includes both a cationic membrane and an anionic membrane. **(D)** Structure of Measurement of resistance of electrolytes in Table S2. Experimental details in Supporting Information 7.

**Figure S14 | Equivalent circuit of artificial action potential chip and power device**

**(A)** Hodgkin-Huxley model for excitable cell membrane. **(B)** Equivalent circuit of ion exchange membranes in a double-Donnan system. **(C)** Whole circuit with multimeter and external resistance. The top line of the circuit is the equivalent circuit of an artificial action potential chip or power devices. **(D)** Equivalent circuit and schematics of a power device based on the Donnan system.

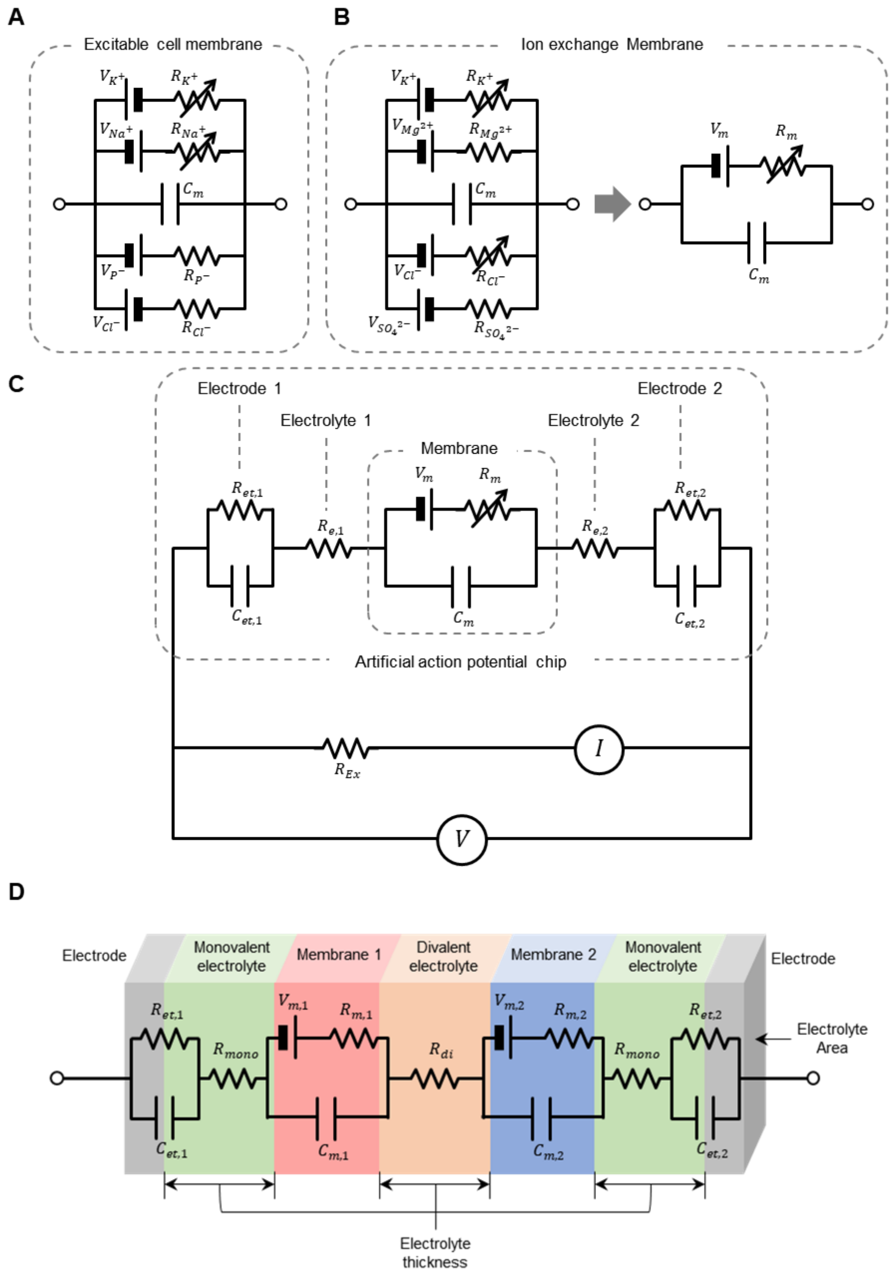

**Table S1 | Ion concentrations and membrane selectivity**

| | $K^+$ | $Na^+$ | $Cl^-$ | $P^-$* | Resting potential | $P_{K^+/Na^+}$ in Resting | Action potential | $P_{Na^+/K^+}$ in Action |
|---|---|---|---|---|---|---|---|---|
| **Squid Axon** | | | | | | | | |
| Extracellular fluid [mM] | 20 [S1] | 440 [S1] | 560 [S1] | -- | -60 mV [S3] | 23.2 | 45 mV [S3] | 15.0 |
| Intracellular fluid [mM] | 400 [S1] | 50 [S1] | 52 [S1] | 385 [S2] | | | | |
| **Mammalian Cell** | | | | | | | | |
| Extracellular fluid [mM] | 4 [S4] | 142 [S4] | 120 [S4] | -- | -70 mV [S5] | 24.1 | 30 mV [S5] | 5.1 |
| Intracellular fluid [mM] | 150 [S4] | 15 [S4] | 5 [S4] | 155 [S4] | | | | |
| **Electrocyte of Electric Eel** | | | | | | | | |
| Extracellular fluid [mM] | 5 [S6] | 172 [S6] | 183 [S6] | -- | -75 mV [S6] | 40.1 | 60 mV [S6] | 45.7 |
| Intracellular fluid [mM] | 170 [S7] | 10 [S7] | Unk** | Unk** | | | | |

| | $K^+$ | $Mg^{2+}$ | $SO_4^{2-}$ | $Cl^-$ | mAEM potential | $P_{Cl^-/SO_4^{2-}}$ | mCEM potential | $P_{K^+/Mg^{2+}}$ |
|---|---|---|---|---|---|---|---|---|
| **Artificial** | | | | | | | | |
| Divalent Electrolyte [M] | -- | 0.5 | 0.5 | -- | - 70 mV | 14.3 | 70 mV | 14.3 |
| Monovalent Electrolyte [M] | 1 | -- | -- | 1 | | | | |

Calculation of relative permeabilities is in Supporting Information 3.

*[$P^-$ : anionic proteins that are impermeable to the cell membrane]

**[Unk : unknown concentration. It is assumed to be similar to the number of counter-polar ions in the same region to satisfy electroneutrality. [S8]]

**Table S2 | Conductivity and resistance of the components of this system**

| | | KCl 0.01M | KCl 0.1M | KCl 1M | MgSO4 0.5M | NaCl 0.5M (Catalog**) |
|---|---|---|---|---|---|---|
| Electrolyte | Conductivity, σ [mS/cm] | 1.59 (1.41[S9]) | 14.25 (12.89 [S9]) | 112.07 (111.73 [S9]) | 33.84 (34 [S9]) | -- |
| | **Area Resistance, $R_e$ [Ω·cm$^2$]*** | 125.61 | 14.04 | 1.78 | 5.91 | -- |
| | Resistance [Ω·cm$^2$], length : 100 um | 6.28 | 0.70 | 0.09 | 0.29 | -- |
| with CEM | $R_e$ +$R_m$ [Ω·cm$^2$] | 130.32 | 17.67 | 2.66 | 8.63 | -- |
| | **$R_m$ [Ω·cm$^2$]** | 4.70 | 3.63 | 0.87 | 2.72 | 1.8 |
| with mCEM | $R_e$ +$R_m$ [Ω·cm$^2$] | 136.79 | 17.57 | 2.74 | 10.66 | -- |
| | **$R_m$ [Ω·cm$^2$]** | 11.17 | 3.53 | 0.96 | 4.75 | 1.8 |
| with AEM | $R_e$ +$R_m$ [Ω·cm$^2$] | 128.89 | 17.61 | 4.27 | 10.08 | -- |
| | **$R_m$ [Ω·cm$^2$]** | 3.27 | 3.57 | 2.48 | 4.17 | 2.6 |
| with mAEM | $R_e$ +$R_m$ [Ω·cm$^2$] | 140.74 | 18.79 | 4.41 | 11.70 | -- |
| | **$R_m$ [Ω·cm$^2$]** | 15.12 | 4.76 | 2.63 | 5.79 | 3.8 |

*[Ω·cm$^2$ : Area resistance is used for easy calculation with membrane resistance, and the electrolyte length is 2 mm and area is in resistance measurement. Experimental details in Supporting Information 7 and 8]

**[Catalog : area resistance listed in Astom Corp.'s catalog.]

**Supporting Information 1 |** Donnan effect in cell membrane and artificial chip

The Donnan effect is an electrochemical phenomenon that occurs in a system with electrolytes and a membrane, when only a specific ion contained in one electrolyte is impermeable to the membrane[S2, S10]. The impermeability of a specific ion with an ion gradient between the two electrolytes affects the behavior of the other ions, both electrically and chemically. In reverse electrodialysis, NaCl electrolytes, which are common brines, are arranged in high and low concentrations, and only chloride ions($Cl^-$) are permeable through the anion exchange membrane, or only sodium ions($Na^+$) are permeable through the cation exchange membrane. For anion exchange membrane(AEM), the arrangement of ions is as follows:

$$\begin{array}{c|c|c} {Na^+}_{High} & & {Na^+}_{Low} \\ {Cl^-}_{High} & \text{AEM} & {Cl^-}_{Low} \end{array}$$

Sodium ions($Na^+$) cannot flow along an ionic gradient. However, when only chloride ions permeat, the electroneutrality of each electrolyte break. This problem is solved by creating an electrical gradient around the membrane to allow sodium ions to overcome the blockage of the membrane while also making it difficult for chloride ions to permeate, even with high membrane permeability. Eventually, owing to the electrical gradient, the two electrolytes maintain electroneutrality, the two ions permeate in the same amount, and the electrical gradient is measured as the membrane potential.

In a two-ions system, the membrane potential assists in the sustained permeation of two ions, which in turn reduces the concentration gradients. The reduction of the concentration gradients is a positive feedback process that reduces the membrane potential; therefore, the discharge of this system cannot be controlled. This problem can be solved by adding impermeable ions as follows:

$$\begin{array}{c|c|c} {Na^+}_{High} & & {Na^+}_{High} \\ {Cl^-}_{High} & \text{AEM}^* & \\ & & {X^-}_{High} \end{array}$$

In this system, the low-concentration NaCl electrolyte was replaced with a high-concentration electrolyte containing sodium ions and another anion $X^-$. The membrane(AEM*) permeated only the chloride ions and not the $X^-$ ions. The permeation of chloride ions must be accompanied by the permeation of sodium ions in the same chamber or $X^-$ ions in the opposite chamber to maintain the electroneutrality of the electrolytes. An electrical gradient is formed around the membrane, which suppresses the permeation of chloride ions and promotes the permeation of two impermeable ions to satisfy the electroneutrality. Unlike in the two-ion system, there was no initial sodium ion concentration gradient. Even the sodium ions permeated with an electrical gradient gradually increased with a concentration gradient opposite to that of the chloride ions. The sodium ion gradient is a negative feedback that suppresses the decrease in the electrical gradient, and only the $X^-$ ion provides positive feedback. If the permeability to sodium ions is higher and only the permeability to $X^-$ ions is low, the effect of inhibiting the decrease in the electrical gradient owing to the increase in the sodium ion gradient may be greater. Therefore, this system could maintain a stable membrane potential.

Even in osmosis, the three-ions system results in less water flow owing to a small concentration difference, and the ion gradient and electric gradient decrease are delayed.

The osmotic pressure is calculated by Van't Hoff equation:

$$\Pi = cRTi$$

where $\Pi$ (Pa) is the osmotic pressure, $c$ $(\mathrm{mol \cdot m^{-3}})$ is the molarity, $R$ $(\mathrm{J \cdot mol^{-1} \cdot K^{-1}})$ is the gas constant, $T$ (K) is the temperature, and $i$ is the Van't Hoff factor.

Some parameters, such as mobility and activity at high concentrations according to ionic species are not considered in the Van't Hoff equation, but a rough estimate is possible. When two electrolytes with the same concentration were used, the osmotic pressure was 0. In the artificial systems,

composed of KCl 1 M and $MgSO_4$ 0.5 M, used to implement the Donnan effect, the osmotic pressure is approximately half the osmotic pressure of the reverse electrodialysis with KCl 1 M and 0.01 M. The osmotic pressure measurement is shown in **Figure S2 A**, and the experimental details are provided in **Supporting Information 4**

In resting, the cell membrane is impermeable to sodium ions($Na^+$), and only potassium ions($K^+$) are permeable, creating a membrane potential(**Figure S1 A** and **B**). To maintain electroneutrality in intra- and extracellular fluid from potassium ion flow, an electrical gradient that inhibits the permeation of potassium ions is generated by surrounding ions[S11, S12]. The electrical gradient also suppresses the permeation of chloride ions($Cl^-$) outside the cell, thereby maintaining the electrochemical equilibrium and electroneutrality. In action, owing to the relatively high permeability of sodium ions, the cell membrane has an opposite electrical gradient that inhibits the inflow of sodium ions(**Figure S1 C**). An electrical gradient in the opposite direction promotes the permeation of chloride ions which have low membrane permeability, thereby satisfying electroneutrality through the same permeation of both ions. The ion composition around the cell membrane based on the Donnan effect helps the homeostasis of the cell and the generation of a membrane potential with low osmotic pressure.

**Supporting Information 2 |** Fabrication and Immunofluorescence staining of tissue-engineered skeletal muscle

Human skeletal muscle myoblasts (HSMM) were purchased from the Lonza. HSMMs were expanded in a T75 flask (Nunc) containing the Skeletal Muscle Cell Growth Medium-2 BulletKit™ (SkGM-2) (Lonza). HSMMs at 80% confluency were dissociated with Accutase (Innovative Cell Technologies) and resuspended in a the hydrogel mixture of rat tail collagen I (Corning) and Matrigel-growth factor reduced (Corning). The HSMM-hydrogel mixture was injected into a rectangular-shaped PDMS chamber, which contained a pair of anchors. HSMMs were incubated for three days with SkGM-2, and the culture medium was changed to differentiation medium consisting of 1:1 mix of DMEM/F12 and Neurobasal supplemented with 1% penicillin-streptomycin, Glutamax, 0.5x N-2, 0.5x B-27 (all from Gibco), and 50 ng/ml IGF-1 (Peprotech). The tissue constructs were cultured for 1-3 weeks and the differentiation medium was exchanged every other day.

The tissue-engineered skeletal muscle constructs were fixed in 4% paraformaldehyde and washed with PBS. Following fixation, the constructs were incubated in PBS containing 3% bovine serum albumin (BSA) (Gibco) and 0.5% Triton-X (Sigma) for 1 hour at room temperature. Tissue samples were incubated with primary antibodies against SAA (Sigma, 1:200 dilution) and MyHC II (Sigma, 1:400 dilution) in 1% BSA and 0.2% Triton-X containing PBS solution for overnight at 4 ℃ After five cycles of PBS washing, the samples were incubated with secondary antibodies (Invitrogen, 1:500 dilution) and 4',6-diamidino-2-phenylindole (DAPI) (Invitrogen, 1:1000 dilution) for 1 hour at room temperature. The tissues were washed with PBS multiple times and imaged using a confocal laser scanning microscope (Zeiss).

**Supporting Information 3 |** Theoretical Background of membrane potential

The flow of ions in an electrolyte is described by the Nernst-Planck equation derived from the law of conservation of mass for charged materials in a fluid. The flux $j_i$ $(\mathrm{mol \cdot m^{-2} \cdot s^{-1}})$ of ion $i$ is affected by the diffusion and electrical forces.

$$j_i = -D_i \left[ \nabla c_i + c_i \frac{n_i F}{RT} \nabla \phi \right]$$

where $D_i$ $(\mathrm{m^2 \cdot s^{-1}})$ is the diffusivity of ion $i$, $c_i$ $(\mathrm{mol \cdot m^{-3}})$ is the concentration of ion $i$, $n_i$ is the valence of ion $i$, $F$ $(\mathrm{C \cdot mol^{-1}})$ is Faraday's constant, and $\phi$ $(\mathrm{V})$ is the electrical potential.

Goldman developed this equation to describe the flow of ions in a system where two electrolytes are separated by a membrane[S13]. In the membrane, the ion current density $J_i$ $(A \cdot m^{-2})$ induced by a specific ionic species $i$ contained in the electrolytes is as follows.

$$J_i = q_i \mu n_i F P_i \frac{[i]_{out} - [i]_{in} e^{n_i \mu}}{1 - e^{n_i \mu}}, \qquad \mu = \frac{F V_m}{RT}$$

where $q_i$ $(\mathrm{C})$ is the charge of ion $i$, $P_i$ $(\mathrm{m \cdot s^{-1}})$ is the permeability of ion $i$ through the membrane, $[i]_{out}$ and $[i]_{in}$ $(\mathrm{mol \cdot m^{-3}})$ are the concentrations of ion $i$ in each electrolyte, and $V_m$ $(\mathrm{V})$ is the membrane potential.

Hodgkin and Katz introduced this equation for the quantitative analysis of membrane potential in excitable cells and compared it with measurements of the actual giant squid axons[S14]. With reference to the composition of ions in the cell membrane that are important for cell metabolism shown in **Table S1**, an equation for the flow of each ion can be established.

$$J_{K^+} = (+1)(+1)\mu F P_{K^+} \frac{[K^+]_{out} - [K^+]_{in} e^{-\mu}}{1 - e^{-\mu}}$$

$$J_{Na^+} = (+1)(+1)\mu F P_{Na^+} \frac{[Na^+]_{out} - [Na^+]_{in} e^{-\mu}}{1 - e^{-\mu}}$$

$$J_{Cl^-} = (-1)(-1)\mu F P_{Cl^-} \frac{[Cl^-]_{out} - [Cl^-]_{in} e^{\mu}}{1 - e^{\mu}}$$

In general, the membrane potential is estimated as the equilibrium potential generated when the flow of all the ions is in equilibrium.

$$J = \sum_i J_i = 0$$

$$V_m = \frac{RT}{F} \ln\left(\frac{P_{K^+}[K^+]_{out} + P_{Na^+}[Na^+]_{out} + P_{Cl^-}[Cl^-]_{in}}{P_{K^+}[K^+]_{in} + P_{Na^+}[Na^+]_{in} + P_{Cl^-}[Cl^-]_{out}}\right)$$

The permeability of anions is low, and there is no change in ion channels related to anions even when generating an nerve signal; therefore, it is ignored for ease of calculation[S15].

$$V_m = \frac{RT}{F} \ln\left(\frac{P_{K^+}[K^+]_{out} + P_{Na^+}[Na^+]_{out}}{P_{K^+}[K^+]_{in} + P_{Na^+}[Na^+]_{in}}\right)$$

$$V_m = \frac{RT}{F} \ln\left(\frac{P_{K^+/Na^+}[K^+]_{out} + [Na^+]_{out}}{P_{K^+/Na^+}[K^+]_{in} + [Na^+]_{in}}\right) = \frac{RT}{F} \ln\left(\frac{[K^+]_{out} + P_{Na^+/K^+}[Na^+]_{out}}{[K^+]_{in} + P_{Na^+/K^+}[Na^+]_{in}}\right)$$

Through this equation, the relative permeability of these two cations in the resting and action states can be determined, as shown in **Table S1**.

On the other hand, conventional reverse electrodialysis systems use two concentrations of NaCl electrolytes and ion exchange membranes. Therefore, the ion flows and membrane potentials can be estimated similarly[S16].

$$J_{\mathrm{Na^+}} = (+1)(+1)\,\mu F P_{\mathrm{Na^+}} \frac{[Na^+]_{out} - [Na^+]_{in} e^{-\mu}}{1 - e^{-\mu}}$$

$$J_{\mathrm{Cl^-}} = (-1)(-1)\,\mu F P_{\mathrm{Cl^-}} \frac{[Cl^-]_{out} - [Cl^-]_{in} e^{\mu}}{1 - e^{\mu}}$$

$$J = \sum_i J_i = 0$$

$$V_m = \frac{RT}{F}\ln\left(\frac{P_{\mathrm{Na^+}}[Na^+]_{out} + P_{\mathrm{Cl^-}}[Cl^-]_{in}}{P_{\mathrm{Na^+}}[Na^+]_{in} + P_{\mathrm{Cl^-}}[Cl^-]_{out}}\right)$$

Common engineered ion exchange membranes are charged with a specific polarity, and the permeability of ions of opposite polarity has been remarkably low in recent research, around 90%[S17]. Therefore, it can be simplified as follows by ignoring anion permeation in a system that selects cations, such as a living body.

$$V_m = \frac{RT}{F}\ln\left(\frac{[Na^+]_{out}}{[Na^+]_{in}}\right)$$

If the membranes do not have large selectivity defects, the potential of the system is highly dependent on the concentration difference. This results in the need for low concentrations.

To generate a large membrane potential even when the total concentration difference between both sides of the membrane is small, such as in a living body, ions of the same polarity with different membrane permeabilities are required. To satisfy this requirement, we used divalent ions and monovalent ion exchange membranes, and the membrane potential and ion flow for this can be described as follows.

In $MgCl_2$ and KCl electrolytes, which are three-ion systems with different cations, the equations are described as follows.

$$J_{\mathrm{Mg^{2+}}} = (+1)(+2)\,\mu F P_{\mathrm{Mg^{2+}}}\frac{0 - [Mg^{2+}]e^{-2\mu}}{1 - e^{-2\mu}}$$

$$J_{\mathrm{K^+}} = (+1)(+1)\,\mu F P_{\mathrm{K^+}}\frac{[K^+] - 0\cdot e^{-\mu}}{1 - e^{-\mu}}$$

$$J_{\mathrm{Cl^-}} = (-1)(-1)\,\mu F P_{\mathrm{Cl^-}}\frac{[Cl^-]_{out} - [Cl^-]_{in}\cdot e^{\mu}}{1 - e^{\mu}}$$

$$J = \sum_i J_i = 0$$

$$V_m = \frac{RT}{F}\ln\left(\frac{[K^+]P_{\mathrm{K^+}} + ([Cl^-]_{out} - [Cl^-]_{in})P_{\mathrm{Cl}}}{2([Mg^{2+}]P_{\mathrm{Mg^{2+}}} + [Cl^-]_{in}P_{\mathrm{Cl^-}})} \pm \frac{\sqrt{\{[K^+]P_{\mathrm{K^+}} + ([Cl^-]_{out} - [Cl^-]_{in})P_{\mathrm{Cl^-}}\}^2 + 4(2[Mg^{2+}]P_{\mathrm{Mg^{2+}}} + [Cl^-]_{in}P_{\mathrm{Cl^-}})([Cl^-]_{out}P_{\mathrm{Cl^-}} + [K^+]P_{\mathrm{K^+}})}}{2(2[Mg^{2+}]P_{\mathrm{Mg^{2+}}} + [Cl^-]_{in}P_{\mathrm{Cl^-}})}\right)$$

When the equivalents of both cations are the same, that is, the chloride ions are the same in both electrolytes, $Cl^-$ permeation can be neglected as follows:

$$V_m = \frac{RT}{F}\ln\left(\frac{P_{\mathrm{K^+}} + \sqrt{{P_{\mathrm{K^+}}}^2 + 8([Mg^{2+}]/[K^+])P_{\mathrm{Mg^{2+}}}P_{\mathrm{K^+}}}}{4([Mg^{2+}]/[K^+])P_{\mathrm{Mg^{2+}}}}\right)$$

$$V_m = \frac{RT}{F}\ln\left(\frac{P_{\mathrm{K^+}} + \sqrt{{P_{\mathrm{K^+}}}^2 + 4P_{\mathrm{Mg^{2+}}}P_{\mathrm{K^+}}}}{2P_{\mathrm{Mg^{2+}}}}\right) = \frac{RT}{F}\ln\left(\frac{P_{\mathrm{K^+/Mg^{2+}}} + \sqrt{{P_{\mathrm{K^+/Mg^{2+}}}}^2 + 4P_{\mathrm{K^+/Mg^{2+}}}}}{2}\right)$$

It can be arranged in the same way in a three-ion system with $K_2SO_4$ and KCl electrolytes that differ only in the anion.

$$V_m = \frac{RT}{F}\ln\left(\frac{P_{\mathrm{Cl^-}} + \sqrt{{P_{\mathrm{Cl^-}}}^2 + 4P_{\mathrm{SO_4{}^{2-}}}P_{\mathrm{Cl^-}}}}{2P_{\mathrm{SO_4{}^{2-}}}}\right) = \frac{RT}{F}\ln\left(\frac{P_{\mathrm{Cl^-/SO_4{}^{2-}}} + \sqrt{{P_{\mathrm{Cl^-/SO_4{}^{2-}}}}^2 + 4P_{\mathrm{Cl^-/SO_4{}^{2-}}}}}{2}\right)$$

In the $MgSO_4$ and KCl electrolytes, which is a 4-ion system to acquire the membrane potential for anions as well, the equations are described as follows.

$$J_{\mathrm{Mg^{2+}}} = (+1)(+2)\,\mu F P_{\mathrm{Mg^{2+}}}\frac{0 - [Mg^{2+}]e^{-2\mu}}{1 - e^{-2\mu}}$$

$$J_{\mathrm{SO_4{}^{2-}}} = (-1)(-2)\,\mu F P_{\mathrm{SO_4{}^{2-}}}\frac{0 - [{SO_4}^{2-}]e^{2\mu}}{1 - e^{2\mu}}$$

$$J_{K^+} = (+1)(+1)\,\mu F P_{K^+} \frac{[K^+] - 0 \cdot e^{-\mu}}{1 - e^{-\mu}}$$

$$J_{Cl^-} = (-1)(-1)\,\mu F P_{Cl^-} \frac{[Cl^-] - 0 \cdot e^{\mu}}{1 - e^{\mu}}$$

$$J = \sum_i J_i = 0$$

$$V_m = \frac{RT}{F}\ln\left(\frac{([K^+]P_{K^+} - [Cl^-]P_{Cl^-}) \pm \sqrt{([K^+]P_{K^+} - [Cl^-]P_{Cl^-})^2 + 4\left(2[Mg^{2+}]P_{Mg^{2+}} + [Cl^-]P_{Cl^-}\right)\left(2[SO_4{}^{2-}]P_{SO_4{}^{2-}} + [K^+]P_{K^+}\right)}}{2\left(2[Mg^{2+}]P_{Mg^{2+}} + [Cl^-]P_{Cl^-}\right)}\right)$$

Under the condition that the monovalent cation exchange membrane is located, the formula can be simplified by ignoring the permeation of the two anions as follows.

$$V_m = \frac{RT}{F}\ln\left(\frac{P_{K^+} + \sqrt{P_{K^+}{}^2 + 8([Mg^{2+}]/[K^+])P_{Mg^{2+}}P_{K^+}}}{4([Mg^{2+}]/[K^+])P_{Mg^{2+}}}\right)$$

If the equivalents of both ions are the same($[Mg^{2+}]/[K^+] = 1/2$),

$$V_m = \frac{RT}{F}\ln\left(\frac{P_{K^+} + \sqrt{P_{K^+}{}^2 + 4P_{Mg^{2+}}P_{K^+}}}{2P_{Mg^{2+}}}\right) = \frac{RT}{F}\ln\left(\frac{P_{K^+/Mg^{2+}} + \sqrt{P_{K^+/Mg^{2+}}{}^2 + 4P_{K^+/Mg^{2+}}}}{2}\right)$$

In the case of a monovalent anion selective membrane, the membrane potential is described in the same manner.

$$V_m = \frac{RT}{F}\ln\left(\frac{P_{Cl^-} + \sqrt{P_{Cl^-}{}^2 + 4P_{SO_4{}^{2-}}P_{Cl^-}}}{2P_{SO_4{}^{2-}}}\right) = \frac{RT}{F}\ln\left(\frac{P_{Cl^-/SO_4{}^{2-}} + \sqrt{P_{Cl^-/SO_4{}^{2-}}{}^2 + 4P_{Cl^-/SO_4{}^{2-}}}}{2}\right)$$

The membrane potential can be estimated from the membrane with known selectivity in the above formula; in this case, the selectivity was estimated based on the measured membrane potentials, as shown in **Table S1**. Previously, the permselectivity of ion exchange membranes was confirmed by including two ions in one electrolyte, confirming the co-ion permselectivity for reverse

electrodialysis. In contrast, this bio-inspired membrane potential confirms counter-ion permselectivity[S18]. It is difficult for the co-ion permselectivity to exceed 2, but the counter-ion permselectivity can be configured with excellent selectivity over 10.

In addition, based on the theoretical equations above, the prediction of the membrane potential of reverse electrodialysis, biologicals and Donnan system under various permselectivity and concentration conditions is shown in **Figure S12**. The membrane potential in each system was calculated using the following equations.

Reverse electrodialysis :

$$V_m = \frac{RT}{F}\ln\left(\frac{P_{\mathrm{Na}^+}[Na^+]_{out} + P_{\mathrm{Cl}^-}[Cl^-]_{in}}{P_{\mathrm{Na}^+}[Na^+]_{in} + P_{\mathrm{Cl}^-}[Cl^-]_{out}}\right)$$

Biologicals : Assume that both cations have the same high-concentration( $[Na^+]_{out} = [K^+]_{in}$ ) and neglect low concentrations.

$$V_m = \frac{RT}{F}\ln\left(\frac{P_{K^+/Na^+}[K^+]_{out} + [Na^+]_{out}}{P_{K^+/Na^+}[K^+]_{in} + [Na^+]_{in}}\right) \approx \frac{RT}{F}\ln\left(P_{K^+/Na^+}\right)$$

Donnan system :

$$V_m = \frac{RT}{F}\ln\left(\frac{P_{\mathrm{K}^+} + \sqrt{{P_{\mathrm{K}^+}}^2 + 8([Mg^{2+}]/[K^+])P_{\mathrm{Mg}^{2+}}P_{\mathrm{K}^+}}}{4([Mg^{2+}]/[K^+])P_{\mathrm{Mg}^{2+}}}\right)$$

For a convenient comparison with the biological membrane potential, potassium ions outside and sodium ions inside the cell membrane were excluded because they have low concentrations. The membrane potential of the cell is:

$$V_m = \frac{RT}{F}\ln\left(\frac{[Na^+]_{out}}{P_{K^+/Na^+}[K^+]_{in}}\right) = \frac{RT}{F}\ln\left(\frac{P_{Na^+/K^+}[Na^+]_{out}}{[K^+]_{in}}\right)$$

Since the concentrations of potassium ions inside the cell and sodium ions outside the cell are similar, if we consider them to be the same, then

$$V_m = \frac{RT}{F}\ln\left(\frac{1}{P_{K^+/Na^+}}\right) = \frac{RT}{F}\ln\left(P_{Na^+/K^+}\right)$$

In the equation of the system using divalent ions, the term in the square root is greater than the selectivity; consequently, the term in the logarithm is greater than the selectivity.

$$V_m = \frac{RT}{F}\ln\left(\frac{P_{\mathrm{K^+/Mg^{2+}}} + \sqrt{{P_{\mathrm{K^+/Mg^{2+}}}}^2 + 4P_{\mathrm{K^+/Mg^{2+}}}}}{2}\right) > \frac{RT}{F}\ln\left(P_{\mathrm{K^+/Mg^{2+}}}\right)$$

$$V_m = \frac{RT}{F}\ln\left(\frac{P_{\mathrm{Cl^-/SO_4{}^{2-}}} + \sqrt{{P_{\mathrm{Cl^-/SO_4{}^{2-}}}}^2 + 4P_{\mathrm{Cl^-/SO_4{}^{2-}}}}}{2}\right) > \frac{RT}{F}\ln\left(P_{\mathrm{Cl^-/SO_4{}^{2-}}}\right)$$

This means that excluding divalent ions with larger charges has a greater effect on membrane potential generation. Therefore, even if the selectivity value is the same, it can have a greater effect on the generation of the membrane potential, depending on the valence of the excluded ion. As shown in **Table S1**, the calculated selectivity was lower in the artificial system than in biological cells, but the resting potentials and artificial membrane potential were similar.

**Supporting Information 4 |** Measurement of osmotic pressure

The osmotic pressure was measured using a larger capacity H-shaped cell. Each reservoir had a wide bottom with a capacity of 100 mL. The upper part is a narrow cylinder with a diameter of 6 mm, with a millimeter scale that allows for checking the capacity change[S19]. The ion-exchange membrane was clamped with silicone rubber between the two reservoirs. The diameter of the passage through the ion-exchange membrane was 8 mm. Cation exchange membranes(CSE, CIMS) were used for osmotic pressure measurements. CSE was used when only KCl electrolytes are used, and CIMS was used when $MgSO_4$ electrolyte was used. Each reservoir was filled with an electrolyte that generated a membrane potential, a stirring bar was placed in it, and the change was observed in capacity while stirring for 25 hours(**Figure S2 A**). The first five hours were excluded because of fluctuations in the internal and external temperature adjustment and degassing effects.

Through the results of osmotic water flow under each ionic condition, it is possible to predict the decrease in membrane potential in a device with a specific volume**. Figure S2 B** shows the analysis result at an effective membrane area of 0.5 $mm^2$ and an electrolyte thickness of 2 mm. In that volume, the number of cations and anions in KCl 1M electrolyte are $10^{-6}$ mol each. In the osmosis experiment of **Figure S2 A**, each electrolyte has a very large volume compared to the effective membrane area, the water flow for 24 hours is around 1% of the initial volume, and the two electrolytes are in a disconnected state with no electrodes connected. Therefore, assuming that there is no significant difference between the number of ions and the volume at the beginning, the average water flow rate for 24 hours can be regarded as the osmotic pressure at the corresponding concentration difference. On the other hand, the shape used in the analysis has a small volume(1 $mm^3$) compared to the effective membrane area(0.5 $mm^2$), so the concentration of each electrolyte continuously changes, and the water flow must be corrected accordingly.

$$\Pi_{in} = (c_{left,in} - c_{right,in})RTi$$

$\Pi_{in}$, $c_{left,in}$, and $c_{right,in}$ are the initial osmotic pressure, the initial electrolyte concentration on the left side of the membrane, and the initial electrolyte concentration on the right side of the membrane.

Since the concentration of the low-concentration electrolyte increases due to the decrease in volume by water flow and the concentration of the high-concentration electrolyte decreases due to the increase in volume, the concentration difference continuously decreases and thus the osmotic pressure and water flow also decreases.

$$\Pi = \frac{(c_{left} - c_{right})}{(c_{left,i} - c_{right,i})}\,\Pi_{in}, \qquad water\ flow\ \mathrm{f} = \frac{(c_{left} - c_{right})}{(c_{left,i} - c_{right,i})}\,\mathrm{f}_{in}$$

The membrane potential over time shown in **Figure S2 C** can be predicted through the ion concentration at each time as shown below.

For reverse electrodialysis systems :

$$V_m = \frac{RT}{F}\ln\left(\frac{P_{\mathrm{K}^+}[K^+]_{out} + P_{\mathrm{Cl}^-}[Cl^-]_{in}}{P_{\mathrm{K}^+}[K^+]_{in} + P_{\mathrm{Cl}^-}[Cl^-]_{out}}\right)$$

For Donnan systems :

$$V_m = \frac{RT}{F}\ln\left(\frac{P_{\mathrm{K}^+} + \sqrt{{P_{\mathrm{K}^+}}^2 + 8([Mg^{2+}]/[K^+])P_{\mathrm{Mg}^{2+}}P_{\mathrm{K}^+}}}{4([Mg^{2+}]/[K^+])P_{\mathrm{Mg}^{2+}}}\right)$$

**Supporting Information 5 |** Theoretical analysis of the discharge process

The analysis of the discharge was performed with different central electrolyte thicknesses based on an effective membrane area of 0.5 mm$^2$ ( $A$ ) and an electrolyte thickness of 2 mm ( $l$ ) as shown in **Figure S4 A**.

First, in the Donnan system, the membrane potential due to the ions migrated at each time is calculated as follows. Unlike Supporting Information 3 and 4, ion flow occurs and each electrolyte contains both cations, so this is taken into account.

$$J_{\mathrm{Mg}^{2+}} = 2\,\mu F P_{\mathrm{Mg}^{2+}} \frac{[Mg^{2+}]_{in} - [Mg^{2+}]_{out} e^{-2\mu}}{1 - e^{-2\mu}}$$

$$J_{\mathrm{K}^{+}} = \mu F P_{\mathrm{K}^{+}} \frac{[K^{+}]_{in} - [K^{+}]_{out} \cdot e^{-\mu}}{1 - e^{-\mu}}$$

$$V_m = \frac{RT}{F} \ln\left(\frac{([K^+]_{in}P_{\mathrm{K}^+} - [K^+]_{out}P_{\mathrm{K}^+}) \pm \sqrt{([K^+]_{in}P_{\mathrm{K}^+} - [K^+]_{out}P_{\mathrm{K}^+})^2 + 4\left(2[Mg^{2+}]_{out}P_{\mathrm{Mg}^{2+}} + [K^+]_{out}P_{\mathrm{K}^+}\right)\left(2[Mg^{2+}]_{in}P_{\mathrm{Mg}^{2+}} + [K^+]_{in}P_{\mathrm{K}^+}\right)}}{2\left(2[Mg^{2+}]_{out}P_{\mathrm{Mg}^{2+}} + [K^+]_{out}P_{\mathrm{K}^+}\right)}\right)$$

Membrane potential is shown in **Figure S4 C**.

In the case of resistance, the Donnan system maintained the resistance value at the initial condition by referring to **Table S2** because the change in resistance was not large due to the high concentration of both electrolytes. This allows the current density to be calculated and is shown in **Figure S4 A**. In the case of concentration change, the number of ions passing through the membrane can be known through the change in charge due to current flow. After subtracting this from the initial number of ions, and including the water flow due to osmosis described in Supporting Information 3, the concentration was calculated through the changed volume and number of ions.

$$i = \frac{V_m}{R}, \qquad \Delta q = i \cdot t, \qquad \Delta c = \frac{F \cdot \Delta q}{A \cdot l}$$

Concentration changes are shown in **Figure S4 B**. Only the reduction of the initial ions of the two electrolytes is indicated, and the reduced ion amount is added to the counter electrolyte. That is, in

the KCl electrolyte, $K^+$ and $Cl^-$ decreased, and $Mg^{2+}$ and $SO_4^{2-}$ were added. Ions added to each electrolyte are not indicated for readability.

In the case of a reverse electrodialysis system, the membrane potential is calculated as:

$$V_m = \frac{RT}{F} \ln\left(\frac{P_{\mathrm{K}^+}[K^+]_{out} + P_{\mathrm{Cl}^-}[Cl^-]_{in}}{P_{\mathrm{K}^+}[K^+]_{in} + P_{\mathrm{Cl}^-}[Cl^-]_{out}}\right)$$

In order to know the current density in this case, the change in resistance has to be considered primarily due to the large concentration difference between the two electrolytes, as shown in **Table S2**.

$$conductivity, \sigma\ [\mathrm{mS/cm}] = -32.301c^2 + 144.22c + 0.151$$

This calibration equation accurately calculates the conductivity at the three concentrations, and through this, the resistance of the electrolyte at various concentrations can be determined. Therefore, the calculated current density is shown in **Figure S4 D**. The current determines the change in the number of ions in the same way as in the Donnan system, and the resulting change in concentration and membrane potential are shown in **Figure S4 E, F**.

**Supporting Information 6 |** Measurement of power density

In the inner graph of **Figure 2E**, the gross power density is the ideal power density in the circuit through which a given current flows. It was calculated from the current and voltage obtained from the i-v curve.

$$P = VI$$

The zero-current voltage $V_m$ (V) measured in the i-v curve is the membrane potential and open-circuit voltage, which is the maximum output voltage of the system. The zero-voltage current $I_{0V}$ (A) is the maximum output from a circuit without a resistor, and is called the short-circuit current. This represents the internal resistance $R_{in}$ (Ω) of the system.

$$R_{in} = \frac{V_m}{I_{0V}}$$

When an external resistance $R_{ex}$ is connected, the power output from the system to the external resistor is as follows.

$$P_{out} = V_{ex} I_{ex}$$

The voltage $V_{ex}$ (V) and current $I_{ex}$ (A) applied to the external resistor were calculated using Ohm's law.

$$I_{ex} = \frac{V_m}{R_{in} + R_{ex}}, \qquad V_{ex} = \frac{R_{in}}{R_{in} + R_{ex}} V_m$$

Therefore, the power applied to the external resistor is as follows.

$$P_{out} = \frac{R_{in}}{(R_{in} + R_{ex})^2} {V_m}^2$$

The maximum power is when the derivative of the above equation is zero.

$${R_{in}}^2 - {R_{ex}}^2 = 0, \qquad R_{in} = R_{ex}, \qquad P_{max} = \frac{1}{4}\frac{{V_m}^2}{R_{in}} = \frac{1}{4} I_{0V} V_m$$

The maximum power of the optimized power device in **Figure 4D** and **E** was calculated as described above.

The actual power density of the power devices was calculated from the current measured in the circuit connected to an external standard resistor(**Figure S14 C**).

$$P = {I_{ex}}^2 R_{ex}$$

The power density measurements at various ionic compositions are shown in **Figure 4C** and **Figure S8.** The maximum power density of the Donnan system is generated in the KCl 4 M and $MgSO_4$ 2 M condition which has the same equivalent of monovalent and divalent electrolytes, respectively(**Figure S8 A**). This is because the electrophoretic force in the opposite direction induced by the gradient of the counter-charged ions is minimized. In reverse electrodialysis using only KCl, as shown in **Figure S8 B**, the maximum power density is generated because of the high electrical conductivity of the 40-fold gradient, where the membrane potential is lower than that of the 400-fold gradient, but the conductivity of the low-concentration electrolyte is higher. This shows a trade-off between conductivity and the potential for reverse electrodialysis.

**Supporting Information 7 |** Equivalent circuit and output current

In addition to the Goldman equation used to estimate the membrane potential or selectivity, the output power of this system also depend on the conductivity of the two electrolytes, including the membrane. It is necessary to configure the equivalent circuit of this system in order to obtain a more accurate output current. Hodgkin and Huxley presented an equivalent circuit and quantitative model considering the electrical properties of the cell membrane to estimate the current and the potential of the excitable cell(**Figure S14 A**)[S20].

In **Figure S14 A**, the ion gradient of each ion inside and outside the cell is a difference in chemical potential, and the ions, which are charged particles, have a corresponding electrical potential difference in an equilibrium state. Therefore, in this model, the electrical potential difference induced by the ion gradients is converted to a cell(battery) ($V_{K^+}$, $V_{Na^+}$, $V_{Cl^-}$, and $V_{P^-}$) which generates voltage. These ion gradients have different degrees of contribution to the overall potential difference of the system depending on the permeability and the selectivity for each ion of the membrane causing this difference is converted into resistors $R_{K^+}$, $R_{Na^+}$, $R_{Cl^-}$, and $R_{P^-}$. The layer of ions around the membrane, which is the component that generates this overall potential difference, is represented by the charges charged in a capacitor with capacitance $C_m$.

As shown in **Figure S14 B**, our work can also be expressed in a similar model using only different types of ions. Considering the ionic composition different from that of the living body, it is composed of the batteries of $V_{K^+}$, $V_{Mg^{2+}}$, $V_{Cl^-}$, and $V_{{SO_4}^{2-}}$, and the resistors of $R_{K^+}$, $R_{Mg^{2+}}$, $R_{Cl^-}$, and $R_{{SO_4}^{2-}}$. Consequently, this model can be simplified to a membrane resistance $R_m$, a cell with voltage $V_m$, and a capacitor with capacitance $C_m$, as shown on the right side of **Figure S14 B**.

On the other hand, the two electrolytes adjacent to the membrane are represented by resistors $R_{e,1}$ and $R_{e,2}$ adjacent to the membrane model in the circuit. In addition, the electrodes required for connection with external resistances or measuring devices are converted into a Randle circuit[S21] and are shown in **Figure S14 C**. In general, the contact between the electrode and electrolyte should be

considered a capacitive property($C_{et,1}$, $C_{et,2}$); however, in this work, using Ag/AgCl electrodes and electrolytes containing chloride ions, this effect is negligible, so it is considered as a simple resistor $R_{et,1}$ and $R_{et,1}$. Therefore, the output current of this system is estimated by considering the internal resistance $R_{in}$ to which these resistors are connected in series.

$$R_{in} = R_{et,1} + R_{el,1} + R_{mem} + R_{el,2} + R_{et,2}$$

We measured the resistance of each component, as shown in **Table S2**, and the detailed method is shown in **Supporting Information 8,** which shows that the electrical resistances of commercial ion exchange membranes are very low compared to those of electrolytes.

Theoretically, the conductivity $\sigma$ $(\mathrm{S \cdot cm^{-1}})$ of the electrolyte per unit area and per unit length is as follows.

$$\sigma = \sum q_i c_i u_i$$

where $u_i$ $(\mathrm{S \cdot cm^2 \cdot mol^{-1}})$ is the mobility of ion $i$ and the other parameters are the same as those in the Goldman equation. Conventional reverse electrodialysis has a limitation in that it has a lower power density than other eco-friendly and biocompatible energy systems; one of the main causes of this is the presence of low-concentration electrolytes. The conductivity of the electrolyte depends on the concentration, and conventional systems generate an electrical potential only with a difference in concentration. The conductivity is very low because of the need for a low-concentration electrolyte. A system based on the Donnan effect can completely solve this problem.

**Supporting Information 8 |** Measurement of resistance of electrolytes and membranes

An electrolyte was filled in a reservoir with a length of 2 mm and area of 0.5 $mm^2$, and an Ag/AgCl electrode of the same area was placed at the end of the reservoir. The Ag/AgCl electrode enables direct electron transfer between the electrolyte and electrode through a redox reaction with a low standard electrode potential, minimizing the influence of the capacitor role in the Randle circuit model. Resistance measurements were performed using the electrochemical impedance spectroscopy(EIS) mode of a VersaSTAT 3 potentiometer. Nyquist plots were obtained by scanning from 1 MHz to 1 mHz with a voltage magnitude of 100 mV. A value that intersects the real axis of the graph is adopted as the resistance of the electrolyte and converted into conductivity and area resistance. To measure the resistance of the membrane, the membrane, it was fixed between two reservoirs identical to the electrolyte resistance measurement. The resistances of the membranes were calculated by subtracting the resistance of the electrolyte from the measured resistance, as shown in **Table S2**. It was confirmed that there was no major problem with the measured resistance through several references[S9].

**Supporting Information References**


[S1] A. L. Hodgkin, W. A. Rushton, *Proc. R. Soc. Med.* **1946**, 134, 444.

[S2] F. G. Donnan, *Chem. rev.* **1924**, 1, 73.

[S3] K. S. Cole, J. W. Moore, *J. Gen. Physiol*. **1960**, 44, 123.

[S4] A. V. Melkikh, M. I. Sutormina, *J. Theor. biol.* **2008**, 252, 247.

[S5] C. L. Stanfield, *Principles of human physiology*, Pearson Higher Ed., Upper Saddle River, NJ, USA **2012**.

[S6] T. B. H. Schroeder, A. Guha, A. Lamoureux, G. VanRenterghem, D. Sept, M. Shtein, J. Yang, M. Mayer, *Nature* **2017**, 552, 214.

[S7] E. Schoffeniels, *Ann. NY Acad. Sci.* **1959**, 81, 285.

[S8] O. S. Andersen, in *Encyclopedia of Metalloproteins*, Cellular Electrolyte Metabolism. Springer, New York, NY, USA **2013**, 580.

[S9] Analytical, R. *Conductance data for commonly used chemicals*, Emerson Process Management, Irvine, CA, USA **2010**.

[S10] F. G. Donnan, *J. Membr. Sci.* **1995**, 100, 45.

[S11] B. Hille, *Ionic Channels of Excitable Membranes*, Sinauer Associates, Sunderland, MA, USA **2001**.

[S12] W. D. Stein, T. Litman, Channels, Carriers, and Pumps: An Introduction to Membrane Transport, Academic Press, San Diego, CA, USA **2014**.

[S13] D. E. Goldman, *J. Gen. Physiol.* **1943**, 27, 37.

[S14] A. L. Hodgkin, B. Katz, *J. Physiol.* **1949**, 108, 37.

[S15] A. L. Hodgkin, A. F. Huxley, *J. Physiol.* **1952**, 116, 449.

[S16] F. G. Helfferich, *Ion Exchange*, Courier Corporation, North Chelmsfold, MA, USA **1995**

[S17] P. E. Długołęcki, A. Gambier, K. Nijmeijer, M. Wessling, *Environ. Sci. Technol.* **2009**, 43, 6888.

[S18] T. Luo, S. Abdu, M. Wessling, *J. Membr. Sci.* **2018**, 555, 429.

[S19] J. Kim, S. E. Lee, S. Seo, J. Y. Woo, C. S. Han, *J. Membr. Sci.*, **2019**, 592, 117394.

[S20] A. L. Hodgkin, A. F. Huxley, *J. Physiol.* **1952**, 117, 500.

[S21] B. E. Conway, in *Electrochemical Supercapacitors: Scientific Fundamentals and Technological Applications,* Electrochemical capacitors based on pseudocapacitance, Springer, New York, NY, USA **1999**, 221.
